# Membrane Molecular Species Remodeling as a Signature of ω-3 Fatty Acid Action in Cultured Neural Cells

**DOI:** 10.1080/17590914.2026.2644959

**Published:** 2026-03-22

**Authors:** Kyndall R. Nicholas, Hennrique Taborda Ribas, Kevin D. Browne, Kamryn R. Purpura, Peining Xu, Ashika Mani, Elizabeth N. Krizman, Clementina Mesaros, D. Kacy Cullen

**Affiliations:** a Department of Neuroscience, Perelman School of Medicine, University of Pennsylvania, Philadelphia, Pennsylvania, USA; b Center for Brain Injury & Repair, Perelman School of Medicine, University of Pennsylvania, Philadelphia, Pennsylvania, USA; c Center for Neurotrauma, Neurodegeneration & Restoration, Corporal Michael Crescenz Veterans Affairs Medical Center, Philadelphia, Pennsylvania, USA; d Biomolecular Mass Spectrometry Core, Center of Excellence in Environmental Toxicology, University of Pennsylvania, Philadelphia, Pennsylvania, USA; e Department of Systems Pharmacology and Translational Therapeutics, Perelman School of Medicine, University of Pennsylvania, Philadelphia, Pennsylvania, USA; f Biostatistics Analysis Center, University of Pennsylvania, Philadelphia, Pennsylvania, USA; g Department of Bioengineering, School of Engineering & Applied Science, University of Pennsylvania, Philadelphia, Pennsylvania, USA

**Keywords:** Astrocytes, brain lipids, docosahexaenoic acid, fatty acid/transport, glycerophospholipids, lipidomics, lipids, membrane remodeling, molecular species, neurons

## Abstract

Omega-3 polyunsaturated fatty acids (ω3 PUFAs) are critical structural components of neuronal membranes, yet the molecular specificity of their incorporation within neural cells remains incompletely defined. We integrated untargeted and targeted lipidomics with lipid ontology analysis and coarse-grained membrane simulations to characterize remodeling in primary rat cortical neurons and neuron–astrocyte co-cultures following supplementation with docosahexaenoic acid (DHA), eicosapentaenoic acid (EPA), or docosapentaenoic acid (DPA). Each ω3 PUFA produced a distinct lipidomic signature. DHA showed the most consistent incorporation, selectively enriching phosphatidylethanolamine (PE) species—particularly PE(18:0/22:6) and PE(18:1/22:6)—associated with membrane curvature and organelle organization. Ontology analysis linked DHA supplementation to intrinsic curvature–related membrane features, and membrane simulations demonstrated enhanced collective bilayer bending without substantial changes in overall membrane thickness. EPA preferentially increased EPA-containing PE species without elevating DHA levels, whereas DPA effects were variable and culture-dependent, indicating selective metabolic handling of individual ω3 species. Differences between neurons and neuron–astrocyte co-cultures underscore the importance of cellular context in ω3-driven remodeling. By resolving ω3 incorporation at molecular species resolution and linking compositional changes to predicted membrane behavior, this study provides a structural framework for understanding how dietary ω3 fatty acids may influence neuronal membrane organization and cellular resilience.

## Introduction

A significant portion of the central nervous system (CNS) is comprised of lipids, which are essential for cellular structure, myelin sheath formation, cell signaling, and overall CNS health (Bruce et al., 2021). Fatty acids, the structural unit of lipids, are critical for development and play a crucial role in the formation of the lipid bilayer of cells, regulating what enters and exits the cell (Mallick et al., 2021). Additionally, fatty acids contribute to the structure of the cellular membrane, providing fluidity and stability. Approximately 50% of the brain is made of lipids, with 35% of these lipids being omega-3 polyunsaturated fatty acids (ω3 PUFAs) (Dighriri et al., 2022). ω3 PUFAs are known as “essential fatty acids” indicating that they need to be obtained through diet, as the mammalian body cannot synthesize them. Over the last century, changes in dietary habits, specifically the Western diet, have led to an increase in saturated fatty acids (SFA) and omega-6 polyunsaturated fatty acids (ω6 PUFAs), with a deficiency in ω3 PUFAs (Emma et al., 2022). ω3 PUFA deficiency is associated with altered immune and inflammatory responses (Labrousse et al., 2018), cognitive dysfunctions (Agrawal & Gomez‐Pinilla, 2012), and the development of psychiatric disorders such as bipolar disorder, depression, and schizophrenia (Lange, 2020).

As a result, ω3 PUFA supplementation has become more prevalent. However, the cellular-level effects of such supplementation, particularly within the brain, remain poorly understood. ω3 PUFAs are fatty acids with multiple double bonds in which the first carbon-carbon double bond is separated from the terminal methyl end of the fatty acid chain by three carbons (Leng et al., 2018). This structural component contributes to its distinct functionalities, such as helping to reverse and reduce inflammation while preserving homeostasis, thereby improving cognitive performance and hemoglobin oxygen saturation. Additionally, ω3 PUFAs protect against neurodegeneration and neuronal death by combatting reactive oxygen species and downregulating the expression of apoptotic proteins while upregulating the expression of antiapoptotic proteins (Dighriri et al., 2022). The benefits of ω3 PUFAs are well established, but the potency of these benefits vary depending on the specific type of ω3 PUFA, cell type, and site of action (Rapoport, 2003).

The plasma membrane is the cell’s first line of defense by maintaining cell homeostasis and function through permeability regulation, transport, signal transduction, exocytosis, and endocytosis (Wood & Sun, 2009). Docosahexaenoic acid (DHA, 22:6), the most abundant ω3 PUFA in the brain, accounts for 40% of the ω3 PUFAs in neuronal tissue and is highly enriched in the neuronal plasma membrane (Dighriri et al., 2022). DHA is the result of multiple elongations and desaturations of the ω3 PUFAs eicosapentaenoic acid (EPA, 20:5) and docosapentaenoic acid (DPA, 22:5) (Dyall, 2015). Early radiotracing studies demonstrated that fatty acids cross the blood brain barrier and are incorporated into specific membrane phospholipids (Baker & Chang, 1980). DHA has higher levels of incorporation into the membrane, while EPA undergoes multiple, extensive, and rapid beta-oxidations once it has crossed the blood-brain-barrier (BBB) (Chen et al., 2013). The structure of these PUFAs has a direct effect on their incorporation and utilization, given that EPA, DPA, and DHA cross the BBB at the same rate and have comparable effects on membrane thickness (Dyall, 2015). The specific membrane glycerophospholipids into which the ω3 PUFAs are incorporated is important, as each glycerophospholipid differs in structure, distinguishing its contribution to membrane dynamics. Specifically, DHA and DPA have an increased incorporation into the membrane phosphatidylethanolamine (PE) and phosphatidylserine (PS), while EPA is enriched in phosphatidylinositol (PI) (Dyall, 2015). The former glycerophospholipids are more abundant than the latter (Wood & Sun, 2009) and provide a potential reason for why the abundance of DHA in the brain is approximately 300 times more than EPA (Dyall, 2015). Furthermore, each phospholipid is accompanied by its own proteins, which are responsible for the cell’s response to external signals, selective transport, and oxidative stress (Mallick et al., 2021).

The kinetics of DHA uptake and turnover into the brain *in vivo* in rodents, non-human primates, and humans, specifically into the membrane, are also well established using radio tracing methods (Duro et al., 2023; Fonlupt et al., 1994; Rapoport, 1999, 2001, 2003; Rapoport et al., 2011; Umhau et al., 2009). However, while it is established that ω3 PUFAs are enriched in neuronal membranes, the impact of ω3 PUFA supplementation on the global neuronal lipidome at a molecular species resolution has yet to be determined. This is important given that plasma DHA levels do not reflect brain DHA levels, highlighting the specificity of lipid metabolism in the brain. PUFA turnover and half-life differ whether it is due to deacylation-reacylation or overall metabolic loss and reincorporation from plasma (Rapoport, 2013). *In vivo*, the glial cells in the brain help neurons metabolize lipids, specifically fatty acids. In neuron-astrocyte interactions, astrocytes form lipid droplets, which are a way of storing fatty acids. Typically astrocytes manage oxidative stress by sequestering excess fatty acids and generating antioxidants, which is generally atypical for neurons (Ioannou et al., 2019). Removing excess FAs is crucial as they can become toxic and harm the integrity of the mitochondrial membrane. Astrocytes also upregulate saturated lipids leading to neuronal death in response to injury or disease that may be associated with lipid build-up (Guttenplan et al., 2021). In the current study, we explore how the lipidome of rat primary neurons and neuron-astrocyte co-cultures respond to supplementation with DHA, DPA, and EPA. Specifically, we compared the lipid classes incorporated to highlight the distinction of lipid utilization between neurons alone versus the neuron-astrocyte complex, which could provide more insight into the *in vivo* multicellular environment. For several decades, *in vivo* studies have established that long-chain polyunsaturated fatty acids, including DHA and EPA, cross the blood-brain barrier and are incorporated into brain phospholipid pools with distinct turnover dynamics (Bazinet & Layé, 2014; Chen et al., 2013; Mitchell et al., 2011; Rapoport, 2003). More recently, facilitated transport mechanisms such as Mfsd2a-mediated lysophosphatidylcholine transport have further defined molecular routes of DHA entry into the CNS (Nguyen et al., 2014). However, while bulk incorporation and turnover kinetics are well characterized, the specific molecular glycerophospholipid species preferentially remodeled within defined neural cell systems remain incompletely resolved. Here, we examine ω3-driven lipid remodeling at a molecular species resolution in primary neurons and neuron-astrocyte co-cultures. Recognizing how individual ω3 PUFA supplementation is utilized throughout the cell’s lipidome help to predict how the cell will respond to or be prepared to respond under instances of biological stress such as disease or injury.

## Methods and Materials

All procedures described in this manuscript adhere to the National Institutes of Health Guide for the Care and Use of Laboratory Animals and were approved by the Institutional Animal Care and Use Committees at the University of Pennsylvania (Protocol #807209) and the Michael Crescenz VA Medical Center (Protocol #01865).

### Experimental Design

For all experiments, 3 independent biological replicates were used, each with 4–6 technical replicates for each experimental condition, totaling 12–18 replicates per condition (Supplemental Figure S1). Each biological replicate consisted of independently prepared primary cultures. Biological replicates exhibit inherent variability in their baseline lipid levels, reflecting pre-existing lipidomic differences attributable to natural biological heterogeneity prior to treatment (Supplemental Figure S2) (Ghosh et al., 2021). Therefore, for the discovery phase, one biological replicate was used for multivariate modeling to minimize inter-experiment variance inherent to primary culture systems. The results from discovery were validated in the other 2 independent biological experiments, by relative quantification of the lipids that emerged from the discovery phase. The biological replicates were performed within 1–2 months, and frozen. Lipid extraction was then performed simultaneously, and lipidomics analysis was conducted in a single batch (see further).

**Figure 1.**
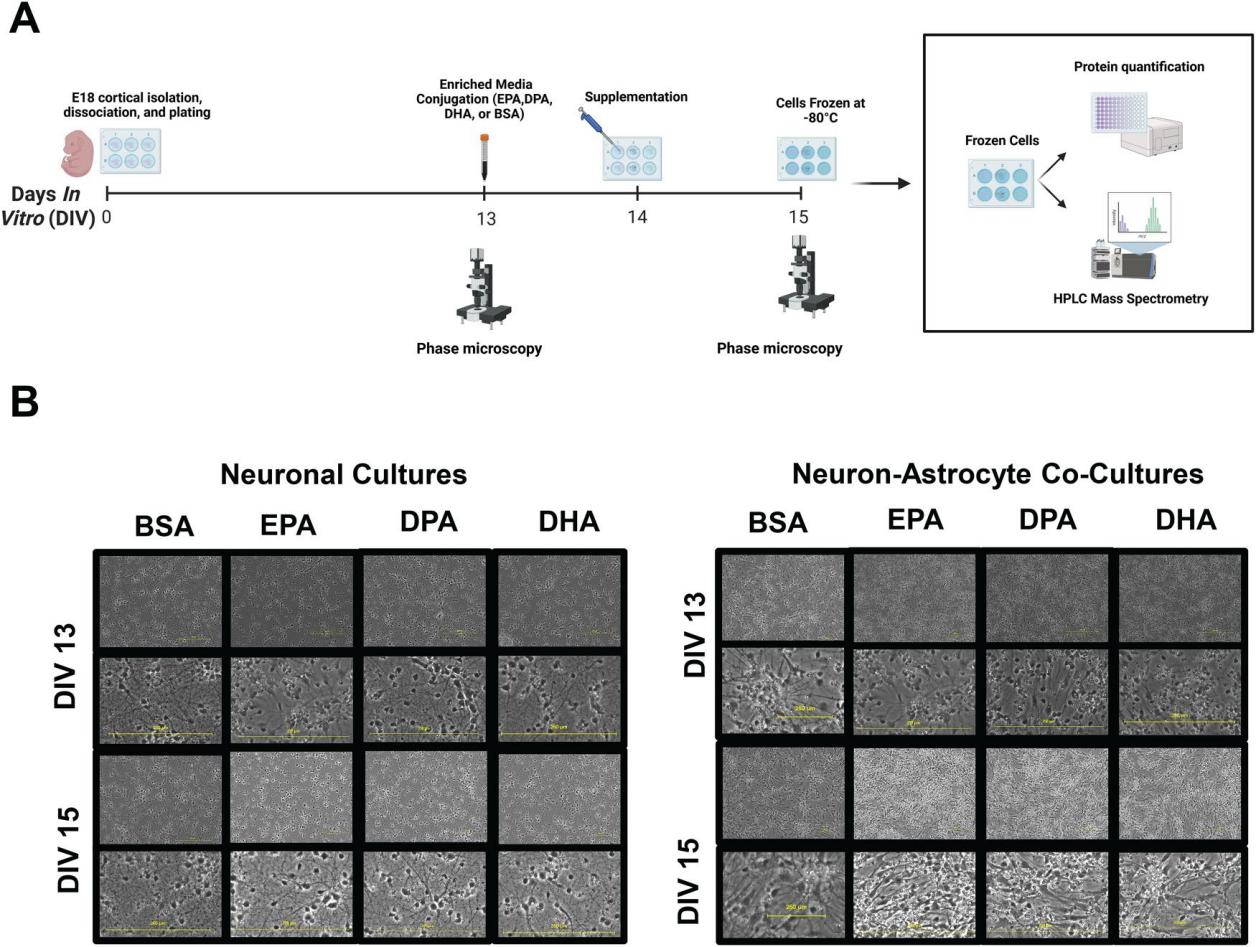
Experimental design, timeline, and cellular morphology following supplementation with EPA, DPA, or DHA. Neuronal cultures and neuron-astrocyte co-cultures were prepared from E18 rat cortices and maintained for 15 days *in vitro* (DIV). On DIV 13, the cultures were imaged on the phase microscope for health and morphology. On DIV 14, the cultures were fed enriched media (25 µM of either EPA, DPA, or DHA) or control media (BSA enriched media). After 24 hours (DIV 15), the cultures were imaged again. The cultures were washed with cold 0.9% saline and frozen at −80 °C for lipidomics. Experiments were conducted in triplicate. (B) Phase microscopy images (10x and zoomed in) from the supplemented cultures 24 hours before (DIV13) and 24 hours after (DIV15) supplementation demonstrated dense healthy cultures of neurons and astrocytes.

Neuronal cultures and neuron-astrocyte co-cultures were prepared and maintained for 15 days *in vitro* (DIV). On DIV 13, the cultures were imaged on the phase microscope for health and morphology; unhealthy cultures characterized as less than 80% confluence and non-adherent cells were discarded. On DIV 14, the cultures were supplemented by enriched media (25 µM of either EPA, DPA, or DHA, each conjugated to BSA) or control media (BSA enriched media). After 24 hours (DIV 15), the cultures were imaged again. The cultures were washed with cold 0.9% saline and flash-frozen at −80 °C for lipidomics analysis (Figure 1).

### Isolation and Culture of Neuronal and Neuron-Astrocyte co-Cultures

Primary cortical neurons and astrocytes were derived from embryonic day 18 (E18) Sprague Dawley embryos of unspecified sex and isolated via microdissection and dissociation using 0.25% trypsin at 37 °C for 12 minutes. The trypsin was deactivated using fetal bovine serum (FBS) (Sigma F0926) and the cell suspension was homogenized via pipette trituration, followed by centrifugation for 3 minutes at 174 x g to create a cell pellet. The trypsin and FBS supernatant were aspirated and replaced with deoxyribonuclease (DNase) (0.15 mg/ml) in HBSS (Invitrogen 14175079). The dissociated tissue and DNase was homogenized via pipette trituration and centrifuged for 3 minutes at 174 g. For neuronal cultures, cells were resuspended in a serum-free media composed of Neurobasal (Invitrogen 21103049), 2% B27 (Invitrogen 12587010), 0.25% Glutamax (Invitrogen 35050061) and 1% penicillin-streptomycin (Invitrogen 15140122) for a final concentration of 500,000 cells per milliliter of media. For co-cultures of neurons and astrocytes, cells were resuspended in a serum-free media composed of Neurobasal, 2% B27, 0.25% Glutamax, 1% penicillin-streptomycin, and 1% G5 for a final concentration of 500,000 cells per milliliter of media. Both types of cultures were plated in 6- well plates (pre-coated with 0.001% poly-l-lysine (Sigma #P4707) and laminin (20 µg/ml) (Corning 354232)) at a density of 500,000 cells per milliliter of media, with 2 milliliters in each well. Three individual experiments were executed where *experiment A* observes DHA supplementation, *experiment B* observes DPA supplementation, and *experiment C* compares DHA supplementation, DPA supplementation, and EPA supplementation. Each experiment consisted of 4–6 wells per media condition, and the experiments were repeated in 3 biological replicates total (Supplemental Figure S1). A half-media change was performed every 3–4 days following plating. At DIV 13, cultures were imaged on the phase microscope (Nikon Eclipse Ti). On DIV 14, half of the media in each well was removed. The same amount of volume removed was replaced by ω3 PUFAs enriched media (50 µM) leading to the final concentration of 25 µM for each PUFA. Twenty-four hours later (DIV 15), the cultures were imaged on the phase microscope.

### Media Preparation for Supplementation

#### ω3 PUFA Media Preparation

A 100 mM stock solution of each ω3 PUFA was prepared in 10 mL of 100% ethanol. Fresh 10% BSA was prepared by dissolving BSA powder (Millipore Sigma A8806) in MilliQ water. 5 mL of 10% BSA was mixed with the 10 mL of the fatty acid solution in a conical tube. The tube was covered with foil and rotated overnight at 37 °C. The following day, the BSA-fatty acid solution (66 mM) was diluted to 11 mM with pre-warmed culture media. The BSA-fatty acid-media solution was covered in foil and stored in 4 °C until ready for use. To prepare the media for supplementation, the BSA-fatty acid-media was prewarmed in 37 °C for 4 hours and vortexed. 5 µL of this solution was mixed into 12 mL of prewarmed culture media to make a 50 µM solution, resulting in 25 µM after the half media supplementation occurred. The final concentration of 25 µM which was determined based on Cao et al. (2005).

#### BSA Media Preparation

BSA media used for control was prepared as described above, without the addition to the fatty acids.

### Lipid Extraction for Lipidomics

At the end of the treatment, each well was washed with 2 mL of cold 0.9% NaCl twice. The plate was then flash-frozen and stored at −80 °C until it was ready for lipid extraction. 1 mL of cold 80% methanol (MeOH) (from −80 °C), 20 µL of SPLASH^®^ LIPIDOMIX^®^ (Avanti Polar Lipids # 330707) diluted 1:1 (v:v) with methanol, and 20 µL (100 ng/µL) of ^13^C_22_-DHA, (Cambridge Isotope Laboratories) were added to each well. The cells were scraped and transferred to an Eppendorf tube on ice. The samples were two round pulse sonicated for 30 seconds and then rested on ice for 10 minutes. The samples were transferred into a 10 mL glass Pyrex tube. The Eppendorf tubes were then rinsed with 200 µL MeOH which was added to the same Pyrex tube. 1.2 mL Optima water was added to each tube, vortexed, and 200 µL of each sample was removed for protein quantification. 100 µL of 0.1% formic acid was added to each sample, followed by 5 mL methyl-tert-butyl-ether (MTBE). Tubes were capped and shaken on mechanical shaker set on “high” for 20 minutes. To induce phase separation, samples were centrifuged for 20 minutes at 600 g with a low acceleration and deceleration to avoid glass cracking. The top layer, containing the lipids in the MTBE/MeOH phase, was transferred into a new 10 mL glass Pyrex tube and evaporated completely under nitrogen gas. The dry residue was resuspended in 200 µL of MTBE/MeOH (1:3 vol ratio). A pooled sample that was used as quality control (QC) was prepared for each batch using 50 µL from each resuspended sample. 100 µL from each sample was then moved to amber glass HPLC vials, with desalinized inserts.

### Lipidomics by Liquid-Chromatography High Resolution Mass Spectrometry (LC-HRMS)

The LC-HRMS method was adapted from our previous protocol (Wang et al., 2021). Lipids were separated using an Accucore C18 HPLC column (2.1 mm x 100 mm, 2.6 µm, # 17126–102130) (Thermo Scientific, Waltham, MA) at 35 °C on an UltiMate 3000 quaternary UHPLC equipped with a refrigerated autosampler (10 °C). Solvent A was 1/1 (v/v) acetonitrile/water with 10 mM ammonium formate and 0.1% formic acid. Solvent B was 10/88/2 acetonitrile/isopropanol/water with 2 mM ammonium formate and 0.02% formic acid. Flow rate was 0.4 mL/min. Flow gradient conditions were as follows: 0 min, 90% A; 1 min, 90% A; 4 min, 60% A; 12 min, 25% A; 21 min, 1% A; 24 min, 1% A; 24.1 min, 90% A; 28 min, 90% A. Samples were analyzed using the QE-HF operated in positive ion mode followed by negative ion mode. 2 µL injections were made. Column effluent was diverted to the QE-HF from 2 to 23 min and to waste for the remaining time of the run.

Data acquisition was performed in Full Scan/ddMS2 mode at 120,000 resolution. The Full Scan settings were as follows: AGC target, 1e6; Maximum IT, 120 ms; scan range, 250 to 1800 m/z. Top 10 MS/MS spectral (dd-MS2) at 15,000 resolution was generated with AGC target = 1e5, Maximum IT = 25 ms, and (N)CE/stepped nce = 25, 30, 35 eV. Mass spec parameters were as follows: spray voltage 4000 V, capillary temperature of 285 °C, probe temperature of 375 °C, sheath gas flow 50 (arbitrary units), auxiliary gas flow 15, sweep gas 1. RF voltage was 45. In the negative mode, the spray voltage was 3500 V, with sheath gas flow set to 55 and auxiliary gas to 20. All other source parameters were optimized to maintain stable spray conditions and consistent ion transmission throughout the analytical run.

### Protein Quantification

The 200 µL saved for protein quantification (see Lipid Extraction for Lipidomics section) were evaporated under nitrogen to dry. 100 µL DPBS was added to each sample, vortexed, and sonicated in a water bath for 5 minutes. Protein quantification was completed using the Pierce 660 nm Protein Assay Reagent (Thermo-Fisher) with a BSA calibration curve constructed in DPBS also. The protein amount from each culture was used for normalization of individual lipids in each well.

### Membrane Simulation

Coarse-grained molecular dynamics simulations were performed using GROMACS 2023.3 (Abraham et al., 2015, 2023) and the MARTINI 2.2 force field (Marrink et al., 2007). Two lipid bilayers with distinct compositions were constructed using the INSANE builder (Wassenaar et al., 2015), each solvated with polarizable MARTINI water and 150 mM NaCl. The control membrane contained POPC:POPE:PUPE at a ratio of 140:57:3, while the DHA-enriched membrane contained **140:54:6**, increasing the proportion of PUPE to model DHA incorporation while maintaining the total lipid number constant. Systems were energy-minimized using the steepest descent algorithm and equilibrated for 50 ns at 310 K using Berendsen temperature coupling (τ_T = 1.0 ps), a 10 fs timestep, and semi-isotropic Berendsen pressure coupling at 1 bar (compressibility 3 × 10^−4 ^bar^−1^, τ_P = 12 ps). Production simulations were run for 20 μs for each membrane, using a 20 fs timestep, velocity-rescale thermostat at 310 K, and semi-isotropic Parrinello–Rahman barostat (1 bar, compressibility 3 × 10^−4 ^bar^−1^, τ_P = 12 ps) (de Jong et al., 2016; Wilson et al., 2020). Analyses of biophysical properties—including area per lipid, membrane thickness, and membrane curvature—were performed using MDAnalysis (Gowers et al., 2016; Michaud‐Agrawal et al., 2011), the Lipyphilic Python package (Smith & Lorenz, 2021), and custom scripts, using GL1/GL2 beads for phospholipids reference positions. Curvature was quantified from triangulated leaflet surfaces using local mean curvature (C), with global curvature represented by the average absolute curvature ⟨|C|⟩ and curvature heterogeneity captured by the distribution width (σ). Visualization of membrane morphology and representative simulation snapshots was performed using PyMOL (The PyMOL Molecular Graphics System, Version 3.0 Schrödinger, LLC).

### Data and Statistical Analysis

For untargeted lipidomics, data analysis was performed with Lipid Search 5 (Thermo-Fisher) using product LC-MS search, with a 0.3 min tolerance for the retention time variability and S/N 5 threshold. For full scan data we used 5 ppm tolerance and 10 ppm for the MS2 data. Lipid species were identified using Lipid Search, and all the exported features were filtered to retain those with a coefficient of variation (CV) < 20% in the pooled QCs that were run through the whole sequence, and a QC/blank intensity ratio > 5. Data were log_2_-transformed prior to univariate analysis, and log_2_-transformed with Pareto scaling for multivariate analysis (PCA and sPLS-PCA) in MetaboAnalyst 6.0 (Idkowiak et al., 2025). Univariate comparisons were performed using two-sided t-tests, and p-values were adjusted for multiple testing using the Benjamini–Hochberg false discovery rate (FDR) method. Further analyses and visualizations were performed in Python using the SciPy, statsmodels, scikit-learn, and matplotlib packages. Lipid species were summed by class to evaluate the abundance of DHA/DPA/EPA-containing lipids, and fold changes between control and treatment groups were calculated to illustrate differential lipid enrichment. When data were transformed into absolute amounts, area for each lipid was normalized by the internal standard from its own class. Afterward, pathway analysis with Lipid Ontology (LiOn) (Molenaar et al., 2019) was used with the filtered and identified lipids with a fold change as identified by Lipid Search 5 and Metaboanalyst. LiOn annotations are inferred biophysical associations based on lipid class and acyl composition, rather than direct experimental measurements.

All analyses for targeted lipidomics were completed using R version 4.1.2 (R Core Team, 2021). We developed a linear mixed-effects model for fatty acid amounts from supplementation. Culture type was included as a fixed effect, and experiment was included as a random intercept to account for between‐experiment variation. Sample name (neuronal versus co-culture) was also included as a fixed effect, and we tested interaction effects between culture type and sample name. We used a linear regression model to analyze PE data using least-squares means, followed by post-hoc pairwise comparisons with Tukey adjustment. Analyses were conducted both within and across culture types to investigate the effect of supplementation on glycerophospholipid amounts.

Experiments were conducted in biological triplicate for each condition and had between 4 and 6 technical replicates depending on the isolated cells for each biological replicate. Since no effect of experiment trial was found based on linear mixed effects model, we randomly selected 4–6 wells for lipidomic analysis.

## Results

### Culture Supplementation with ω3 PUFAs

#### Supplementation with ω3 PUFAs Does Not Alter Cellular Morphology or Density

Cultures using cortical tissue developed multiple neurites per neuron with continuous growth within the first week in culture (Cullen et al., 2010). Neurite outgrowth continued during the second week of culture before supplementation on DIV 14. Neurons were qualitatively assessed across cultures, and experiments showed that supplementation with DHA, DPA, or EPA did not affect cellular morphology or spatial distribution of cell-cell interactions when compared to the control cultures (Figure 1). To identify if ω3 PUFA supplementation altered cell number, protein concentration of each culture was measured. Supplemental Figure S3 shows no significant difference between the DHA or DPA supplementation compared to the control in the same type of cells.

#### DHA Supplementation Increases DHA Levels and Decreases EPA in Neuronal Cultures

**Figure 2.**
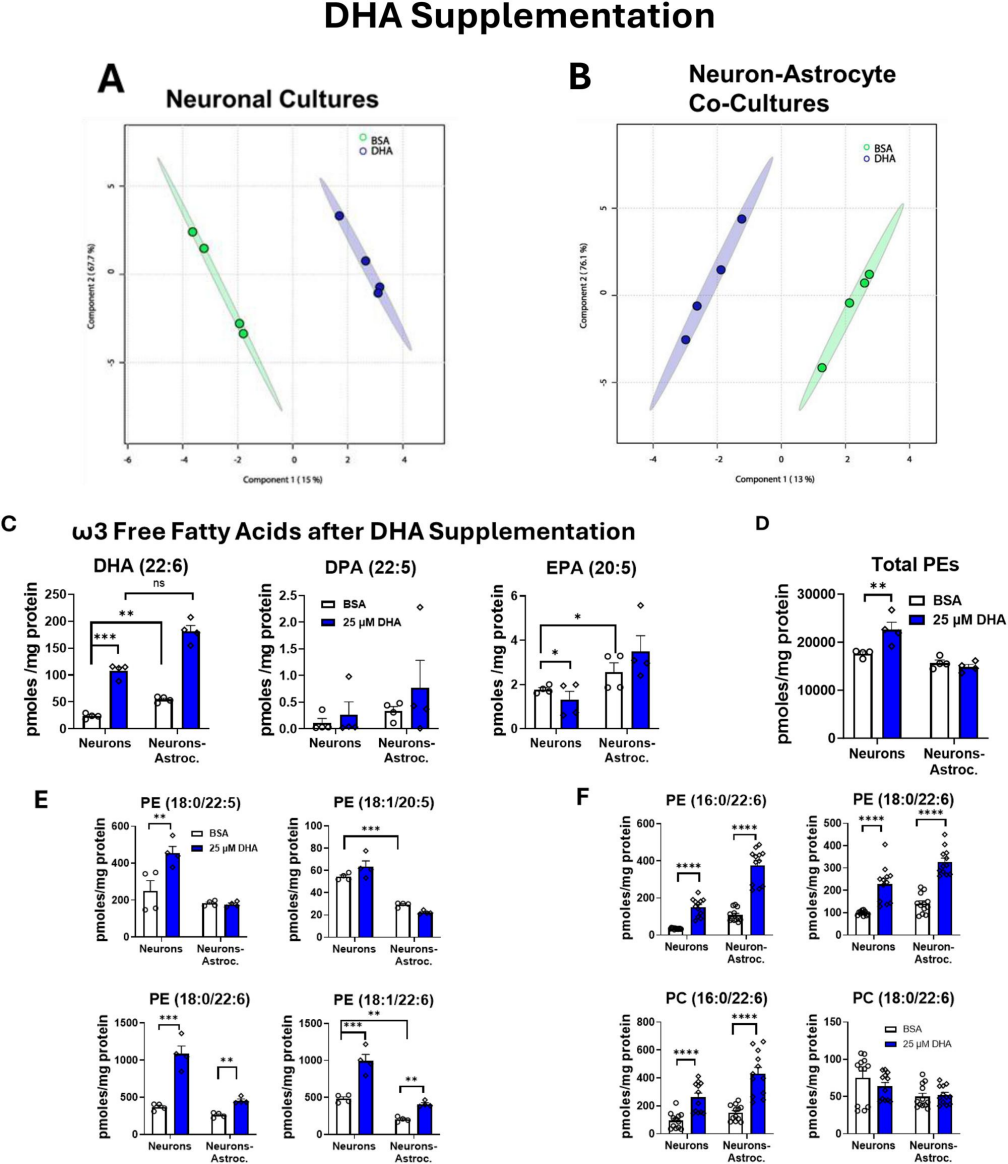
DHA supplementation effects on the cellular lipidome and phosphatidylethanolamines (PEs). Twenty-four hours after supplementation, DHA altered the lipidome of neuron and neuron-astrocyte cultures and increased the number of unique PEs in both culture types and the total amount of PEs in neuronal cultures. (A,B)The sPLS-PCA plots show a clear separation between DHA supplemented cultures and control-treated (BSA) cultures. (C) DHA supplementation increases the amount of DHA and decreases the amount of EPA in neuronal cultures but has no effect on DHA (*p* = 0.5860), DPA, or EPA in co-cultures. ***p ≤ 0.001, **p ≤ 0.01, *p ≤ 0.05; error bars represent SEM. (D) Total amount of Phosphatidylethanolamines increase with DHA supplementation in neurons. (E) Amount of different PEs increase ***p ≤ 0.001, **p ≤ 0.01, *p ≤ 0.05, error bars represent SEM. (F) Validation of the DHA supplementation from all 3 biological experiments. ***p ≤ 0.001, ****p ≤ 0.0001, error bars represent SEM. Samples were normalized prior to analysis by the lipidomic internal standard, followed by BSA protein levels in each culture. (A,B) were analyzed with multivariate analysis. *(C-E)* were analyzed using linear regression model using least-squares means, followed by post-hoc pairwise comparisons with Tukey adjustment. (F) analyzed using Welch’s T test. (A-E) represents data from 1 biological replicate and 4 technical replicates (*N* = 1, *n* = 4) *F* represents 3 biological replicates and 12 technical replicates (*N* = 3, *n* = 12). The trends from the one biological replicate were validated with the other 2 biological replicates but are not visualized due to variability inherent to biological variability.

Unsupervised principal component analysis (PCA) using all the lipids identified after the DHA supplementation (Supplemental Table S1) showed the quality control samples (pooled-QC) tightly grouped in between all the groups (Supplemental Figure S4). We used the untargeted data from only one biological experiment (in this case Exp 1, which had 4 technical replicates), and we went back to validate the findings in the other 2 biological replicates. The pooled QCs were equally distributed between the randomized LC-HRMS runs. For the targeted amounts, the peak area was normalized by the DHA internal standard area (Supplemental Figure S5) and transformed into amount based on the amount of internal standard added, then further normalized to the amount of protein. Further, we used sPLS-PCA (Figure 2A,B) which showed for both neuron-only (Figure 2A) and neuron-astrocyte co-cultures (Figure 2B), there was a clear separation between the cellular lipid profile after DHA supplementation. The amount of DHA in the neuronal cultures significantly increased with supplementation (*p* = 2.17e-13). The level of EPA was about 100 times lower than the DHA levels and decreased as a result of DHA supplementation (*p* = 0.017). DPA levels remained unchanged (Figure 2C). In the co-cultures, the amount of DHA, EPA, and DPA remained unaffected. Interestingly, the basal levels of DHA and EPA (normalized by the amount of protein in each well) were significantly higher in the neuron-astrocyte co-cultures (*p* = 0.002 and 0.010, respectively). DPA levels had a lot of variability between the 3 biological replicates, were much lower than the other two fatty acids and not significantly different among the neuronal culture types tested. These differences may reflect the contribution of astrocyte-derived fatty acids.

#### DHA Supplementation Alters the Number and the Type of Glycerophospholipids

As shown in Supplemental Figure S6, DHA supplementation altered glycerophospholipids regardless of culture type. Triglycerides, diglycerides, and glycerophospholipids that have a 22:6 (DHA) incorporated were increased, including phosphatidylethanolamines (PE), phosphatidylcholines (PC), phosphatidylserines (PS), and phosphatidylglycerols (PG). PE(18:1/22:6), PE(16:0/22:6), PE(18:0/22:6), PE(20:4/22:6), PC(18:0/22:6), PC(16:0/22:6), PS(20:2/22:6), PS(20:4/22:6), and PG(22:5/22:6) were among the glycerophospholipids that increased due to supplementation (Supplemental Figure S6). Total amount of PEs was significantly increased after DHA supplementation in neurons (Figure 2D). The number of PEs containing 22:6 was significantly increased in both culture types (Figure 2E). PE(18:0/22:6) increased in both culture types (*p* < 0.0001 in neurons; *p* = 0.00791 in co-cultures) due to supplementation and while PE(18:1/22:6) was higher in neurons than co-cultures at baseline, but increased with supplementation in both culture types (adjusted *p* < 0.0001 in neurons; *p* = 0.00202 in co-cultures). Supplemental Figure S7 shows the clear increase in the area under the peak for the PE(18:1/22:6) after DHA treatment in neurons, and the HR mass together with the MS2 data was used for identification. The number of PEs incorporating precursors to DHA, such as 20:5 (EPA) and 22:5 (DPA), differed based on the culture type (Figure 2E). In neurons, DHA supplementation significantly increased PE(18:0/22:5) compared to the control neurons (adjusted *p* = 0.0042); however, in the co-cultures, PE(18:0/22:5) did not differ from supplementation (Figure 2E). PE(18:1/20:5) was significantly higher in neurons than co-cultures at baseline (adjusted *p* < 0.0001) but remained unchanged after supplementation regardless of culture type. Yet, DHA supplementation led to a nonsignificant downward trend of PE(18:1/20:5) in co-cultures (*p* = 0.0728). We validated these findings from one experiment’s lipidomics by quantifying two PEs and two PCs in all three biological experiments (Figure 2F). The incorporation of DHA into different PEs was statistically significant, thereby supporting a focused analysis of the PE lipid class.

#### DHA Supplementation Alters Membrane and Cellular Elements

In both culture types, the lipid ontology highlighted DHA supplementation-related alterations in lipids associated with the endoplasmic reticulum, the mitochondrion, membrane components, and negative intrinsic curvature (Supplemental Figure S8). Specifically, lipids associated with above average lateral diffusion, very low bilayer thickness, and lipid droplet and storage were identified in neuronal cultures, while lipids associated with high lateral diffusion and low bilayer were identified in co-cultures.

#### DPA Supplementation Increases DPA, but Not EPA or DHA, Levels in Neuron-Astrocyte Co-Cultures

**Figure 3.**
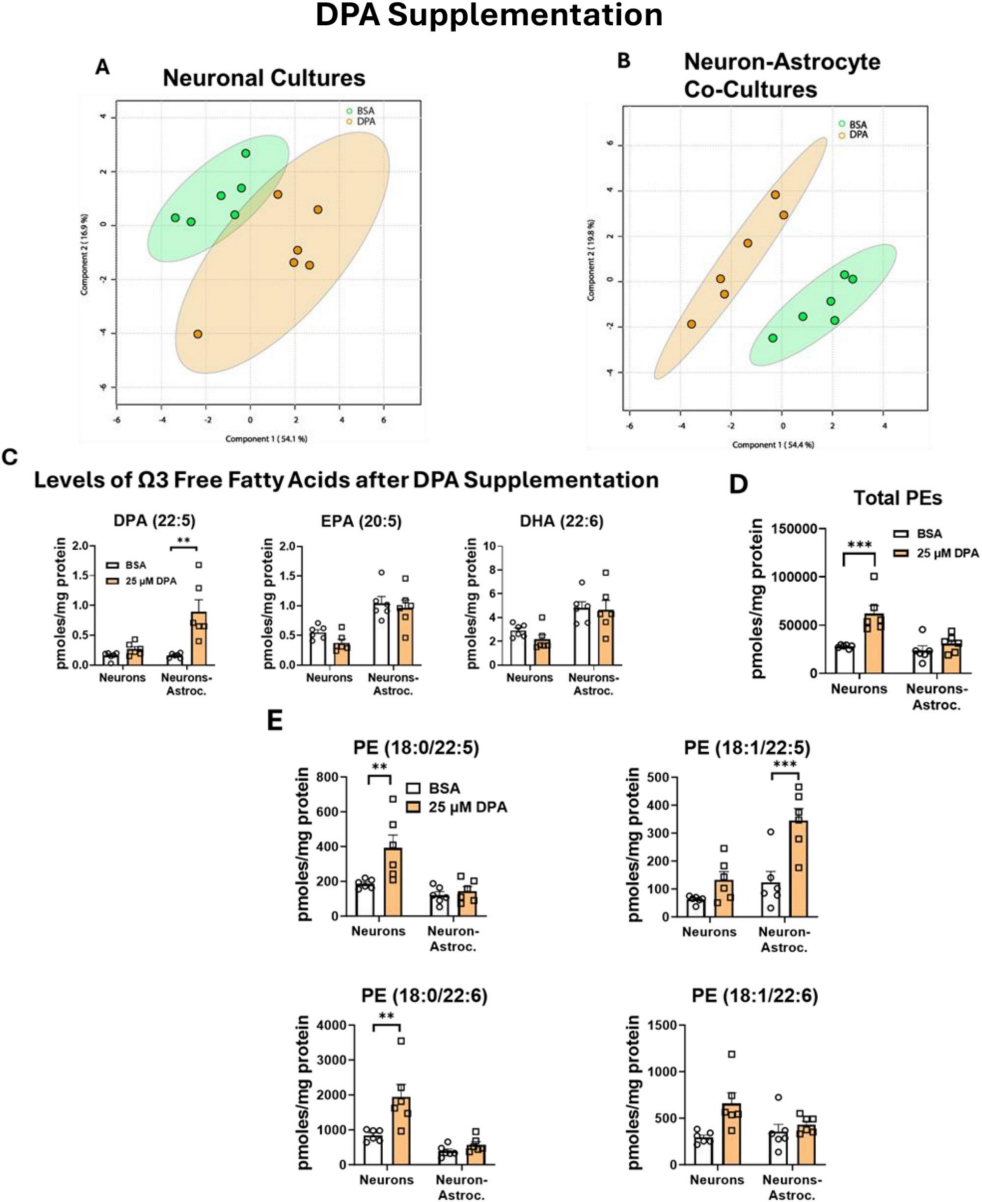
DPA supplementation effects on the cellular lipidome. 24 hours after supplementation, DPA supplementation altered the lipidome of neuron-astrocyte cultures, but not neuronal cultures. DPA supplementation also increases unique PEs in both culture types and the total amount of PEs in neuronal cultures. (A,B) The sPLS-PCA plot visualizing the difference between DPA supplemented cultures and control cultures. (C) DPA trends upward in neuronal cultures and increases in co-cultures. ***p ≤ 0.001, **p ≤ 0.01, *p ≤ 0.05, error bars represent SEM. (D) Total amount of Phosphatidylethanolamines increase with DHA supplementation in neurons. (E) Amount of individual PEs. ***p ≤ 0.001, **p ≤ 0.01, *p ≤ 0.05, error bars represent SEM. Samples were normalized prior to analysis by the lipidomic internal standard, followed by BSA protein levels in each culture. *(A,B)* were analyzed with multivariate analysis. (C-E) were analyzed using linear regression model using least-squares means, followed by post-hoc pairwise comparisons with Tukey adjustment. (A-E) represents data from 1 biological replicate and 6 technical replicates (*N* = 1, *n* = 6). The trends from the one biological replicate were validated with the other 2 biological replicates but are not visualized due to variability inherent to biological variability. This experiment consisted of 3 biological replicates and 18 technical replicates (*N* = 3, *n* = 18).

Unsupervised principal component analysis (PCA) using all the lipids identified after the DPA supplementation (Supplemental Table S2) showed the quality control samples (pooled-QC) tightly grouped in between all the groups (Supplemental Figure S9). We used the untargeted data from only one biological experiment (in this case Exp 1, which had 6 technical replicates), and we went back to validate the findings in the other 2 biological replicates. In neuronal cultures, there was not a clear separation between the cellular lipidomes of the supplemented cultures and the control cultures in the sPLS-PCA (Figure 3A). In contrast, there was a clear separation between the cellular lipidomes of the supplemented co-cultures and the BSA treated co-cultures (Figure 3B), as well as an increase in the level of DPA (*p* = 0.000442) (Figure 3C). Otherwise, there was no statistical difference between supplemented neuronal cultures and supplemented neuron-astrocyte co-cultures, regardless of the ω3 PUFAs analyzed (Figure 3C).

#### DPA Supplementation Alters Glycerophospholipids

While supplementation with DPA led to an increase in the unique glycerophospholipids in both culture types (Supplemental Figure S10), there was a lot of variability in the data. After normalization with the internal standard and the protein amount, only the neuronal cultures increased in the total amount of PEs (adjusted *p* = 0.0008) (Figure 3D). Supplementation of neuron-only cultures resulted in a significant increase in PE(18:/22:5) (adjusted *p* = 0.0088) and PE(18:0/22:6) (adjusted *p* = 0.0034). There appeared to be a trend toward higher levels of PE(18:1/22:6), but this did not reach statistical significance (*p* = 0.0691). In the co-cultures, PE(18:1/22:5) (*p* = 0.0001) was significantly increased (Figure 3E).

#### DPA Supplementation Impacts Membrane and Cellular Elements

According to the lipid ontology (Supplemental Figure S11), both culture types altered lipids associated with glycerophospholipids, membrane components, and the endoplasmic reticulum (Supplemental Figure S11). Alterations in neuronal cultures identified mitochondrion and positive intrinsic curvature. The data presented so far, isolated the effects of DHA and DPA individually compared to BSA-control media and shed light on the baseline effects of these supplementations.

#### EPA, DPA, and DHA Supplementation Increases Respective ω3 PUFAs Levels

Unsupervised principal component analysis (PCA) using all the lipids identified after the three different fatty acids supplementation (Supplemental Table S3) showed the quality control samples (pooled-QC) tightly group in between all the groups (Supplemental Figure S12). We were interested in drawing direct comparisons across EPA, DPA, and DHA supplemented cultures. However, due to spatial limitations of the culture plates and instrumental variability, separate experiments were performed. Accordingly, this experiment evaluated the effects of EPA, DPA, and DHA on cellular lipidomic profiles in neuronal and neuron-astrocyte co-cultures.

**Figure 4.**
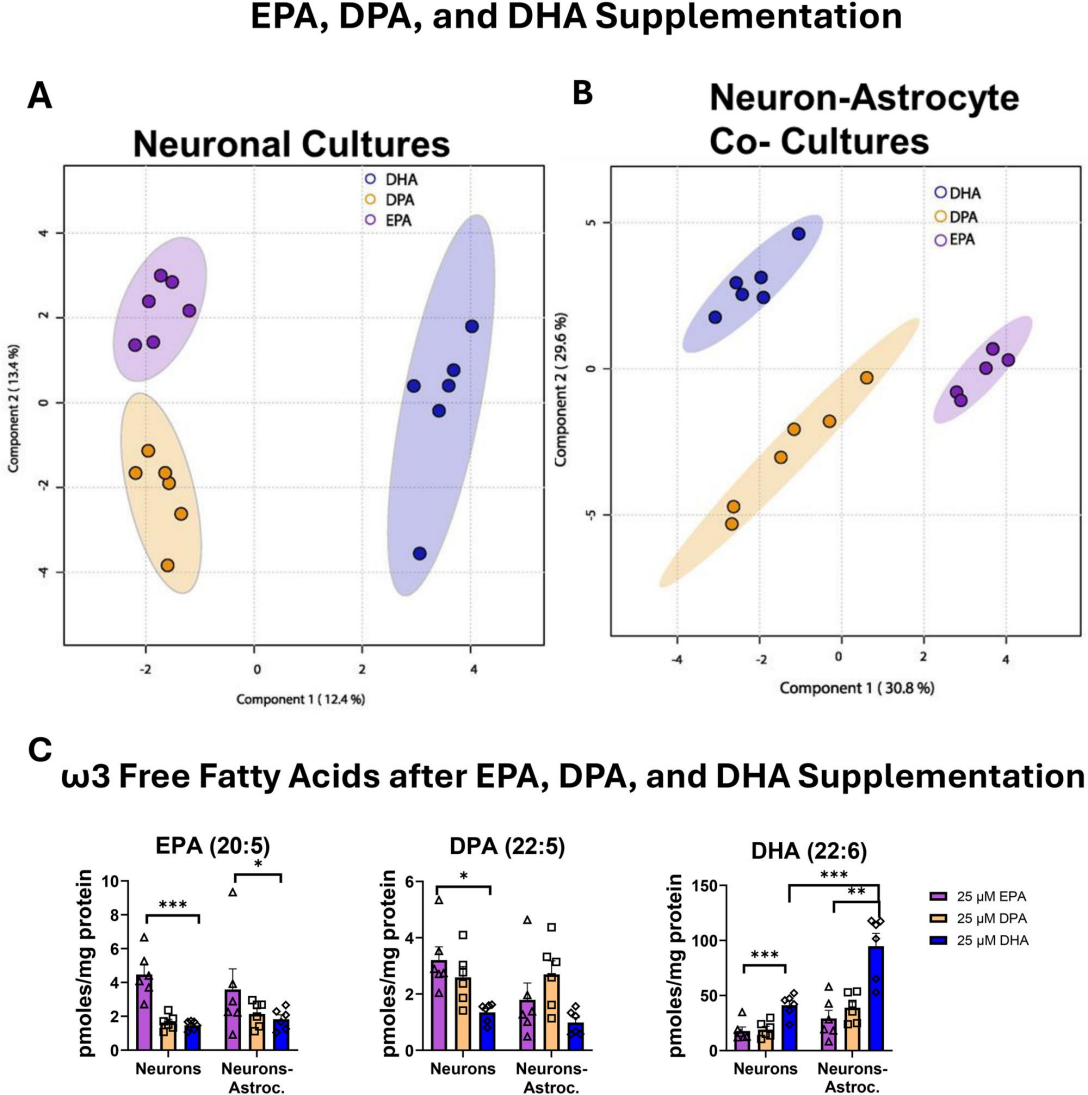
EPA, DPA, and DHA supplementation effects on the cellular lipidome of neuron and neuron-astrocyte cultures after 24 hours. (A,B) The sPLS-PCA plots visualize the difference between supplemented cultures and control cultures. (C) In neurons, EPA supplementation increases EPA, DPA supplementation increases DPA and DHA supplementation increases DHA. In co-cultures, EPA supplementation increases EPA, DPA supplementation trends DPA upward, and DHA supplementation increases DHA. ***p ≤ 0.001, **p ≤ 0.01, *p ≤ 0.05, error bars represent SEM. Samples were normalized prior to analysis by the lipidomic internal standard, followed by BSA protein levels in each culture. (A,B) were analyzed with multivariate analysis. (*C)* was analyzed using linear regression model using least-squares means, followed by post-hoc pairwise comparisons with Tukey adjustment. (A,B) represents data from 1 biological replicate and 6 technical replicates (*N* = 1, *n* = 6). The trends from the one biological replicate were validated with the other 2 biological replicates but are not visualized due to variability inherent to biological variability. This experiment consisted of 3 biological replicates and 16 technical replicates (*N* = 3, *n* = 16).

In both neuron-only and neuron-astrocyte co-cultures, sPLS-PCA showed a clear separation between EPA, DPA, and DHA supplementation (Figure 4A and B). The amount of EPA was significantly higher in EPA supplemented neuronal cultures (*p* = 6.02e-5) and EPA supplemented co-cultures (*p* = 0.025) when compared to the other two supplementations in the respective culture type (Figure 4C). DPA supplemented neuronal cultures showed an increase in DPA when compared to the other two supplementations (*p* = 0.0443), but not in the co-cultures. The amount of DHA was the highest in the DHA supplemented neuronal cultures (*p* = 2.81 e-7 compared to DPA neurons; *p* = 1.50 e-7 compared to EPA neurons) and the DHA supplemented co-cultures (*p* = 0.0124 compared to DPA supplemented neurons; *p* = 0.0022 compared to EPA supplemented neurons) when compared to the other two supplementations in the respective culture type. As observed in the previous experiments, the DHA supplemented neurons had significantly less DHA than the DHA supplemented co-cultures (*p* = 3.9e-5).

#### EPA, DPA, and DHA Supplementation Alter Different Glycerophospholipids

EPA supplementation altered several glycerophospholipids, plasmogens, and triglycerides that incorporated EPA and DPA (Supplemental Figure S13A) in the neuronal cultures, while EPA supplementation primarily altered glycerophospholipids and plasmogens that incorporated EPA in the co-cultures (Supplemental Figure S13B). In the neurons, EPA supplementation was significantly higher in PE(18:1/22:5) (adjusted *p* < 0.0001) and trended upwards in PE(18:2/22:6), although it did not reach statistical significance (adjusted *p* = 0.0737) when compared to DHA supplemented neurons. EPA supplementation was also significantly higher in PE(18:1/20:5) than the other supplemented neurons (adjusted *p* = 0.0002) (Supplemental Figure S14B). There were no significant differences observed in neuronal cultures as a result of DPA or DHA supplementation in the PEs analyzed (Supplemental Figure S14B). In the co-cultures, EPA supplementation was significantly higher in PE(18:1/20:5) than the other two supplementations (*p* = 0.0035). DPA supplemented co-cultures were higher in PE(18:1/22:5) (*p* = 0.0030) and had a trend upward in PE(18:2/22:6) but did not reach statistical significance (*p* = 0.0972) when compared to DHA supplemented co-cultures. DHA supplementation was significantly higher in PE(18:0/22:6) (*p* = 0.0424) than EPA co-cultures and higher in PE(18:1/22:6) (adjusted *p* = 5.22e-5) than the other supplemented co-cultures. Overall, neuronal cultures were significantly higher than the co-cultures in PE(18:0/22:5) and PE(18:0/22:6). When comparing the three supplementations across both culture types, while there were changes in unique glycerophospholipids dependent on the ω3 PUFAs, neither supplementation increased the total amount of PEs (Supplemental Figure S14A).

#### EPA Supplementation Alters Cellular and Membrane Components

When EPA supplementation is compared to DHA supplementation, the lipid ontology identified that in both culture types, EPA altered lipids associated with the endoplasmic reticulum, mitochondrion, glycerophospholipids, and membrane components (Supplemental Figure S15). Lipid alterations in neuronal cultures are associated with positive intrinsic curvature, and lipid storage and droplets, while lipids associated with negative intrinsic curvature were identified in the co-cultures.

### Membrane Simulation of DHA Supplementation

#### DHA Supplementation Alters Membrane Biophysical Properties and Promotes Bilayer Bending in Membrane Simulation

**Figure 5.**
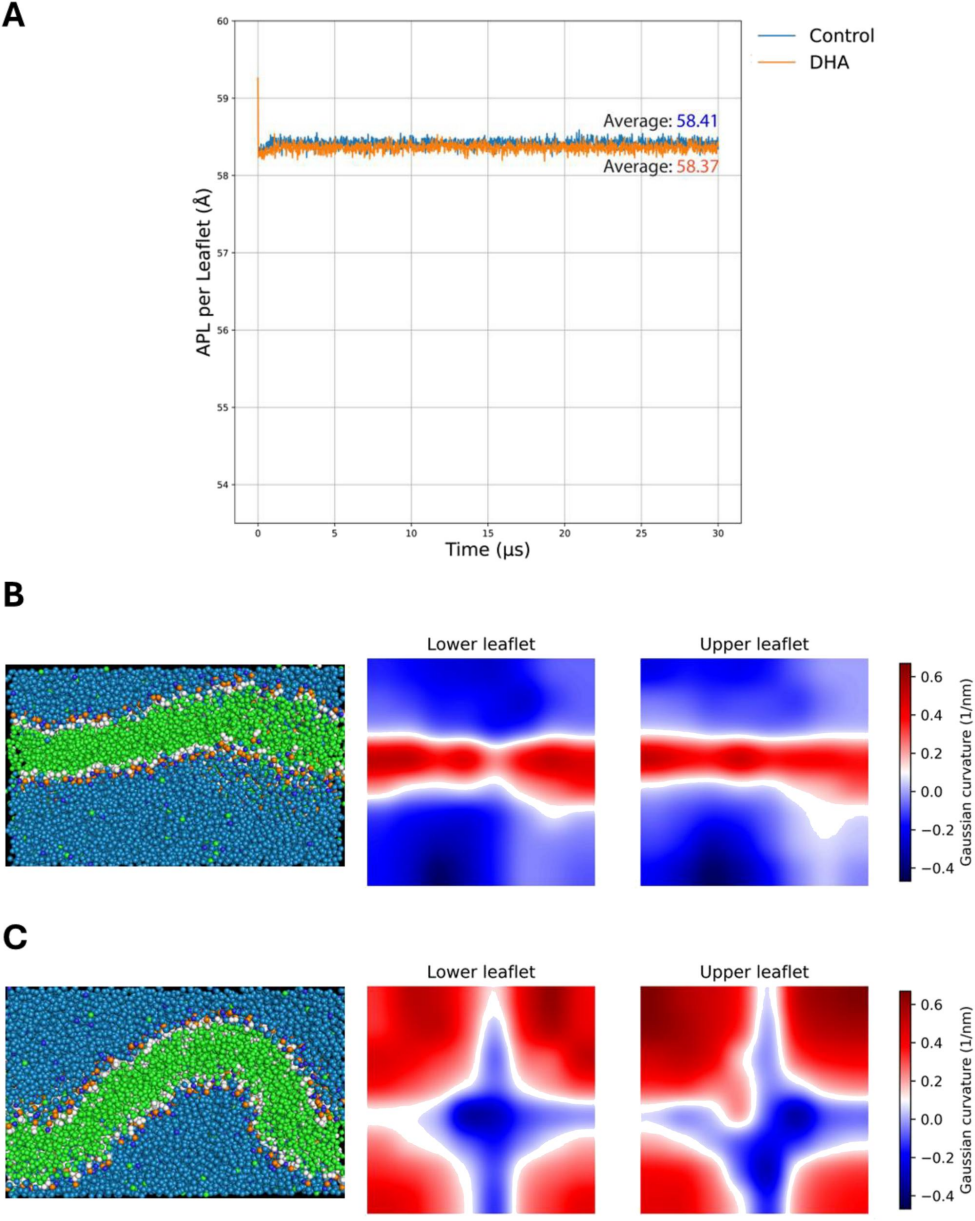
DHA incorporation promotes membrane bending. (A) Area per lipid (APL) per leaflet as a function of simulation time for control and DHA-enriched membranes. Individual traces represent temporal fluctuations, the average APL for each condition are represented, showing no substantial differences between membranes. (B) Control membrane. Left: Representative PyMOL snapshot of the equilibrated bilayer displaying a low-magnitude curvature. Right: Mean curvature map derived from MD analysis showing low-magnitude curvature uniformly distributed across the membrane, consistent with minimal bending. (C) DHA-enriched membrane. Left: Representative PyMOL snapshot illustrating pronounced membrane bending. Right: Corresponding mean curvature map revealing spatially correlated curvature peaks across the bilayer, indicative of coupled leaflet deformation and enhanced bilayer undulation following DHA-PE enrichment.

Due to DHA’s abundance in the brain compared to the other two ω3 PUFAs and the consistency within the lipidomic data, we evaluated our lipidomic output computationally. To evaluate the impact of increased DHA-containing phosphatidylethanolamine (PE) on membrane biophysical properties, we performed coarse-grained molecular dynamics simulations using the MARTINI force field. We compared membrane behavior before and after DHA supplementation to assess changes in mechanical properties (Figure 5). Based on experimental lipidomic data, two membrane compositions were constructed: POPC:POPE:PUPE 140:57:3 (control) and POPC:POPE:PUPE 140:54:6 (DHA supplementation), corresponding to an increase of DHA-containing PE from 5% to 10% of total PE following supplementation.

Analysis of global membrane parameters revealed no substantial differences in area per lipid (APL) or membrane thickness between control and DHA-treated membranes. Given that increased acyl chain unsaturation in PE lipids is known to favor membrane curvature, we next examined membrane bending properties. Notably, DHA-treated membranes exhibited a pronounced increase in bilayer bending compared to control membranes.

Gaussian curvature maps revealed spatially correlated curvature patterns across the upper and lower leaflets of the bilayer. In DHA-containing membranes, regions of positive and negative curvature appeared at matching spatial locations in both leaflets, indicating collective bilayer undulations rather than leaflet decoupling. This behavior was supported by a positive correlation between upper and lower leaflet curvature maps, consistent with enhanced bilayer bending upon DHA incorporation.

## Discussion

In this study, we examined the direct effect of DHA, DPA, and EPA on neurons and neuron-astrocyte co-cultures *in vitro*. Our findings indicate that ω3 PUFAs induce selective lipid remodeling in neurons and neuron–astrocyte co-cultures, revealing distinct cellular preferences for DHA, EPA, and DPA. Among these, DHA produced the most extensive incorporation into membrane phospholipids, notably PE(18:0/22:6) and PE(18:1/22:6), consistent with increased membrane curvature, fluidity, and subcellular organization. Incorporation of EPA and DPA was more limited, highlighting differential metabolic channeling of individual ω3 species in neural cells. Thus, this investigation builds on foundational knowledge about incorporation into membranes but deepens our understanding of incorporation into glycerophospholipids by identifying specific species.

These *in vitro* results extend classic *in vivo* studies (Rapoport, 1999, 2001, 2003; Rapoport et al., 2011), who demonstrated through quantitative imaging that arachidonic acid (ARA) and DHA are maintained in separate, rapidly turning-over phospholipid pools in the brain, each supporting distinct signaling and homeostatic functions. Our data suggest that DHA enrichment reorganizes lipid networks associated with mitochondria and the endoplasmic reticulum, aligning with Rapoport’s model of dynamic DHA turnover and compartmentalized membrane metabolism.

The increase in DHA was not unexpected as it is integral for neuronal function (Weiser et al., 2016). However, we hypothesized that EPA supplementation would increase DHA levels given that EPA is a precursor to DHA (Banaszak et al., 2024). The data show that EPA supplementation did not increase the amount of DHA, which suggests that EPA undergoes beta-oxidation and is involved in other pathways in addition to DHA synthesis, as has been previously demonstrated (Chen et al., 2013). Interestingly, we found that DPA had no effect on DPA levels and had limited effect on all PUFAs levels in the lipidome. The DPA data have more variability than the other two PUFAs supplementation, likely due to it being a precursor for many lipids and its ability to retro-convert (Drouin et al., 2019), making its fate less obvious after supplementation. This pattern likely reflects the distinct metabolic fates of individual ω3 PUFAs: DHA is the preferred structural ω3 fatty acid required for membrane integrity and signaling in CNS cells, whereas EPA is more readily oxidized for energy. DPA, in turn, functions primarily as an intermediate and reservoir that can be converted to either EPA or DHA (Byelashov et al., 2015). Overall, our data demonstrates that the specificity of the ω3 PUFAs contribute to their utilization and implementation.

Our ontology analysis suggests that DHA, DPA, and EPA supplementation led to plasmalemma structural and biochemical changes. Glycerophospholipids were altered by each supplementation in both culture types. Phospholipids in the cell’s membrane serve a unique and important function as each phospholipid is accompanied by its own protein which are responsible for many of the cell’s homeostatic capabilities. The effectiveness of these functions under different environmental conditions is dependent on the membrane’s fluidity (Cooper, 2000), which is directly influenced by ω3 PUFA incorporation (Leng et al., 2018). Specifically in DHA supplementation, the lipid modifications noted in the ontology are associated with membrane dynamics. DHA supplementation led to changes in lipids associated with bilayer thickness and lateral diffusion. Bilayer thickness is inversely proportional to lateral diffusion (Ramadurai et al., 2010), the movement of molecules within the membrane, and negatively correlated with the permeability of the membrane, movement of molecules across the membrane (Kondrashov & Akimov, 2023). Interestingly, a major determinant of lateral diffusion is the viscosity of the plasma membrane (Ramadurai et al., 2010), which is influenced by the head groups of the phospholipids. PEs, a major glycerophospholipid that was altered and modified from supplementation, increase the hydrogen bonds with neighboring molecules in the membrane, thus increasing the viscosity of the membrane and increasing the bilayer thickness. Although alterations in PE composition following supplementation from the membrane simulation did not produce marked changes in bilayer thickness or lateral diffusion, they were sufficient to influence membrane intrinsic curvature. Indeed, our ontology data suggests that membrane dynamics may be affected by PUFA supplementation through membrane intrinsic curvature (Graham & Kozlov, 2010; Jarsch et al., 2016; Kaltenegger et al., 2021).

Curvature analysis revealed a pronounced increase in collective bilayer bending in DHA-enriched membranes. Gaussian and mean curvature maps showed spatially correlated curvature patterns across both leaflets, indicating enhanced bilayer undulations rather than leaflet decoupling. These findings support the experimental observations and suggest that increased DHA-containing PE promotes membrane bending by lowering the energetic cost of curvature deformation.

An alteration in glycerophospholipids suggests a change in the conformation of the plasma membrane. Pure lipid bilayers are flat and alteration of the local lipid composition by changed components such as the lipid head or tail can lead to curvature (Jarsch et al., 2016). Negative intrinsic curvature was highlighted in the neuronal cultures supplemented with DHA, and the co-cultures supplemented with DHA and EPA. Positive intrinsic curvature was highlighted in the neuronal cultures supplemented with DPA and EPA. It is also understood that PEs and the PE-to-PC ratio in the cellular membrane contribute to negative intrinsic curvature due to their conical shape from a smaller headgroup compared to PCs (McMahon & Boucrot, 2015), while positive intrinsic curvature is associated with PIs and LPCs, due to their larger head group and inverted conical shape (McMahon & Boucrot, 2015; Peeters et al., 2022). This is notable as DHA is typically enriched in PEs and EPA is typically enriched in PIs. Our data shows increase in several 22:6 containing PEs, that are crucial in the membrane components and potentially the membrane dynamics of the cells (McMahon & Boucrot, 2015).

Alteration in curvature may indicate changes in the cell’s ability to function and adapt as needed in response to extracellular or intracellular changes. The direction of the relationship between membrane intrinsic curvature and membrane dynamics is not fully established, yet the changes in curvature are evidence of remodeling of the membrane through our supplementation and potential changes to shape related cellular dynamics. While the details of the lipid ontology category, membrane components, are not disclosed, it is possible that the components are referring to the downstream effects of changing the glycerophospholipids and intrinsic curvature, such as membrane fluidity.

In addition to possibly modifying the membrane, supplementation can also alter intracellular components such as the endoplasmic reticulum and the mitochondria, which are both responsible for synthesizing lipids and many structural phospholipids (van Meer et al., 2008). The membrane of the endoplasmic reticulum has been identified as the origin of lipid droplets (Olzmann & Carvalho, 2019), which were also identified as being affected by DHA and EPA supplementation. Lipid droplets store fatty acids as energy rich triglycerides to either remove excess free fatty acids from the cytoplasm, which are toxic and deteriorate the mitochondrial membrane, or to serve as alternative energy during nutrient depletion (Ioannou et al., 2019). While neurons cannot typically do this, neighboring astrocytes will do so in response to oxidative stress. However, lipid droplets can appear in neurons during aging or stress (Cerasuolo et al., 2024). While the concentration used to supplement was selected for its role in enhanced neuronal viability compared to higher concentrations (Cao et al., 2005), the lipid ontology profile is consistent with patterns previously associated with lipid stress response from the excess fatty acids that they could not destroy nor remove themselves. This may provide an explanation for the synthesis of lipids that would typically trigger astrocytes to form and store lipid droplets to prevent lipid toxicity (Zhang et al., 2025) only present in the neuronal cultures. While lipid droplets are rich in triglycerides, lipid rafts, a small membrane domain which provide structural support, are rich in sphingolipids and cholesterol (Cerasuolo et al., 2024). Incorporation of ω3 PUFAs into the membrane directly influences its structure due to their aversion to cholesterol, another structural membrane element (Leng et al., 2018), thereby influencing the formation of lipid rafts. The ω3 PUFA supplementation altered the lipid metabolism of the cells, as seen by the changes in the glycerophospholipids. Research demonstrates that changes in lipid metabolism which affect lipid droplets and lipid rafts are present in neurological diseases and injury (Adibhatla & Hatcher, 2008; Cerasuolo et al., 2024; Sviridov et al., 2020). Lipid droplet and storage was not highlighted in the neuronal cultures supplemented with DPA, which may be due to the variability of the effects of the ω3 PUFAs, emphasizing the individuality of effects by the specific type of ω3 PUFA. Given that astrocytes are responsible for removing and utilizing the free fatty acids in the cytoplasm, the lack of this category in the lipid ontology for the co-cultures furthers the importance of astrocytes and may elucidate the response of neurons to ω3 PUFAs in the absence of astrocytes or in the presence of dysfunctional astrocytes. Additionally, the lipid metabolism of neurons and astrocytes is coupled to maintain a healthy nervous system (Zhang et al., 2025). Astrocytes metabolize excessive free fatty acids and provide phospholipids to support neuronal growth, while neurons utilize the astrocyte-derived support to offset any functional limitations. These glial cells specifically secrete phosphatidic acid (PA) which influence the phospholipid composition of neuronal membranes. Interestingly, PA is the precursor for the synthesis of major glycerophospholipids such as PE, PS, PC, PG, diglycerides, and triglycerides (Carman & Henry, 2007). Following PA in the mitochondrial phosphatidylserine decarboxylase pathway, PE species with polyunsaturated fatty acids are synthesized (Calzada et al., 2016). Our findings are consistent with the possibility that incorporation of PUFA-enriched PE species is augmented through neuron–astrocyte coupled lipid metabolism. Although the exact contributions of either the neuron or astrocyte remain undefined, integration of the established literature with our data supports the possibility that the astrocyte-derived PA could contribute to the synthesis and incorporation of PE into the neuronal membrane. Despite the need for additional validation, the present data provide a focused mechanistic rationale for systematic evaluation of discrete PE species. Based on our observations, there also appeared to be potential differences in the utilization and implementation of ω3 PUFA supplementation in the neuronal cultures versus the neuron-astrocyte co-cultures. The control co-cultures had more DHA and EPA than the control neuronal cultures, DHA supplemented co-cultures had more DHA than the DHA supplemented neurons, the control co-cultures had more DPA than the DPA supplemented neurons, and the EPA supplemented co-cultures had more EPA than the EPA supplemented neurons. Astrocytes can synthesize DHA on their own, potentially contributing to the difference in the ω3 PUFAs in the co-cultures versus the neurons (Williard et al., 2001). The lipid ontology highlighted the difference in the extent of the lipid changes related to the bilayer thickness and lateral diffusion in the co-cultures, as well as the absence of the lipids associated with lipid droplet and storage. Combined, these data highlight the established significance in the presence of astrocytes regarding lipid utilization. Overall, this investigation is in agreement with previous studies on the criticality of functional astrocytes for proper lipid metabolism and function in neurons due to the astrocytes’ ability to synthesize and store lipids (Barber & Raben, 2019; Ioannou et al., 2019).

The findings from this investigation underscore how unique ω3 PUFAs elicit distinct responses on the lipidome of CNS cells. Understanding these differences provides the foundation for lipid therapeutics addressing the cellular response under instances of biological stress such as disease or injury.

This study has several limitations. The *in vitro* system lacks systemic lipid transport, blood–brain barrier dynamics, and long-term metabolic adaptation present *in vivo*. The 25 µM supplementation and 24-hour exposure represent a controlled enrichment paradigm designed to capture early remodeling events rather than steady-state brain physiology. Additionally, cultures were derived from embryos of unspecified sex, precluding evaluation of sex-dependent differences in lipid metabolism. Despite these limitations, the experimental design allowed isolation of ω3-specific remodeling signatures under defined cellular conditions and integration with biophysical modeling to generate testable hypotheses regarding membrane architecture. Furthermore, due to the difficulty of separating neurons and astrocytes without sacrificing the quality of the cells, we did not isolate neurons from astrocytes in the co-cultures to evaluate each cell type’s contribution to the outcomes measured. Rather, we took a “whole culture” approach toward the analysis. This limits our interpretation on how efficiently neurons or astrocytes individually respond to ω3 PUFA supplementation when forming an interactive network. For an in-depth understanding of the specific contributions that astrocytes and neurons bring to the lipid alterations observed, future studies involving cell sorting or isotope tracing could dissect the cell-type specific mechanisms (Cao et al., 2005; Rapoport et al., 2011; Richieri & Kleinfeld, 1995). The *in vitro* system also lacks the lipid buffering system within the selectively permeable blood brain barrier such as the Mfsd2a transporter (Nguyen et al., 2014). Therefore, these limitations preclude direct translational extrapolation, but our data suggests that at a molecular species level, neural cells respond differently when present in an enriched environment with various ω3 PUFAs. Our investigation highlights a link between the remodeling of specific PE species to predicted intrinsic membrane curvature through the integration of LC-HRMS lipidomics, lipid ontology analysis, and coarse-grained membrane simulations. Altogether, these findings build a foundation for the suggested future studies *in vivo* to better interpret the influence of specific PE species in membrane remodeling of the brain after supplementation of ω3 PUFAs.

## Conclusion

The plasma membrane is central to neuronal signaling, structural integrity, and adaptive response to stress. Here, we show that ω3 polyunsaturated fatty acid supplementation produces distinct and species-specific remodeling of the neuronal lipidome, with DHA exerting the most consistent incorporation into phosphatidylethanolamine molecular species linked to membrane curvature and organelle organization. These compositional changes were associated with predicted alterations in intrinsic membrane curvature, supporting a structural role for DHA-enriched PE species in modulating membrane architecture.

Importantly, our findings demonstrate that individual ω3 fatty acids are not metabolically equivalent in neural systems. DHA, EPA, and DPA exhibit differential incorporation patterns and remodeling signatures, reflecting selective cellular handling rather than uniform lipid enrichment. By resolving these effects at molecular species resolution and integrating lipidomics with ontology-based and simulation analyses, this study provides a structural framework for understanding how ω3 supplementation influences neuronal membrane organization.

Together, these data highlight DHA-containing phosphatidylethanolamines as principal mediators of ω3-driven membrane remodeling and establish a foundation for future *in vivo* studies examining how dietary ω3 fatty acids may contribute to membrane dynamics, cellular resilience, and neurological health.

## Supplementary Material

Figure S13.tif

Figure S5.TIF

Figure S10.TIF

Figure S11.TIF

Figure S4.TIF

Figures S1.TIF

Suplemental Table S3_Nicholas EPA DPA DHA exp.xlsx

Figure S12.TIF

Figure S6.TIF

Figure S15.TIF

Suplemental Table S2_Nicholas DPA exp.xlsx

Figure S2.TIF

Figure S9.TIF

Figure S8.TIF

Figure S3.TIF

Figure S14.TIF

Suplemental Table S1_Nicholas DHA exp.xlsx

Figure S7.TIF

## Data Availability

The data that support the findings of this study are available from the corresponding author, C.M., upon reasonable request.

## Abbreviations

DHA: Docosahexaenoic acid
DPA: Docosapentaenoic Acid
EPA: Eicosapentaenoic acid
PA: Phosphatidic Acid
ω3 PUFAs: Omega-3 polyunsaturated fatty acids
