# Supplementary figures and images for: Membrane Molecular Species Remodeling as a Signature of ω-3 Fatty Acid Action in Cultured Neural Cells
Source: ASN Neuro. 2026 Mar 22;18(1):2644959. doi: 10.1080/17590914.2026.2644959 (PMC13007425; doi:10.1080/17590914.2026.2644959)

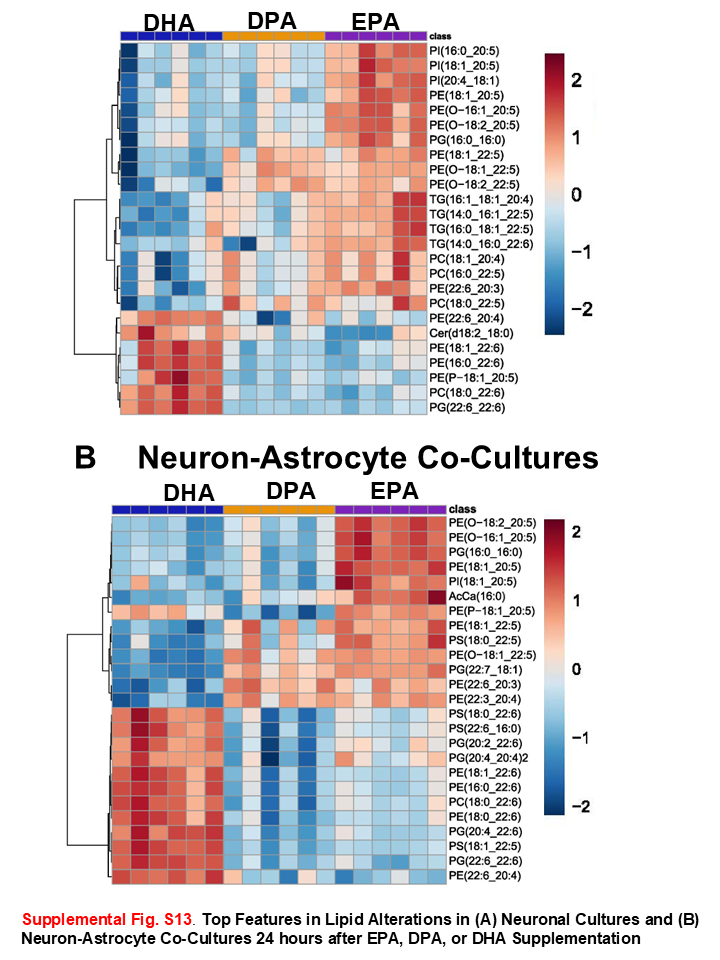

Supplement: Figure S13.tif [file TASN_A_2644959_SM8044.tif]

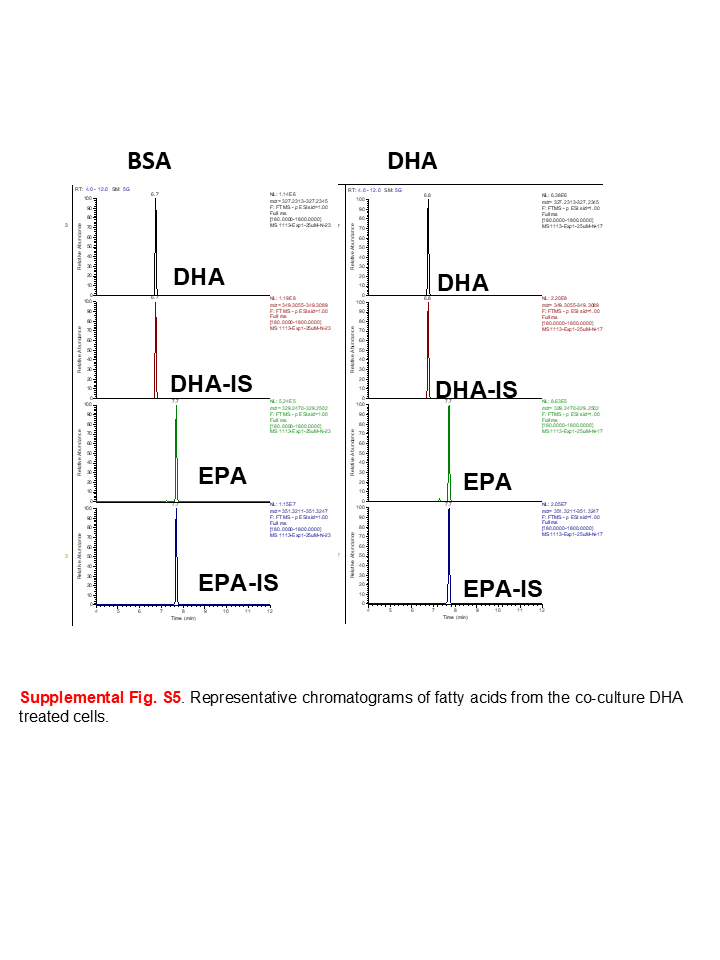

Supplement: Figure S5.TIF [file TASN_A_2644959_SM8043.tif]

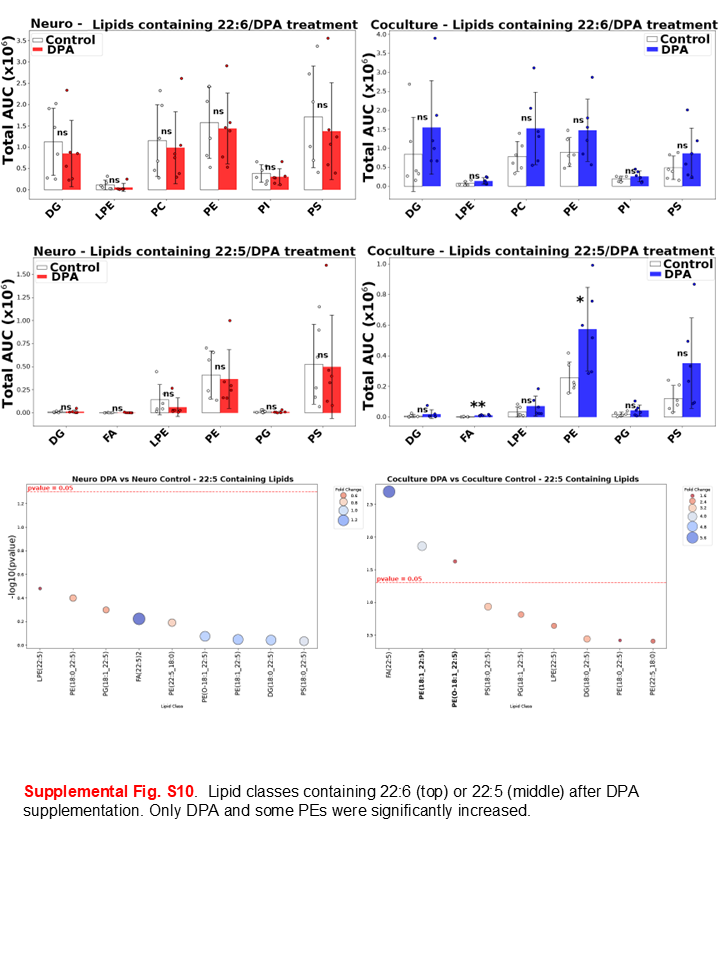

Supplement: Figure S10.TIF [file TASN_A_2644959_SM8042.tif]

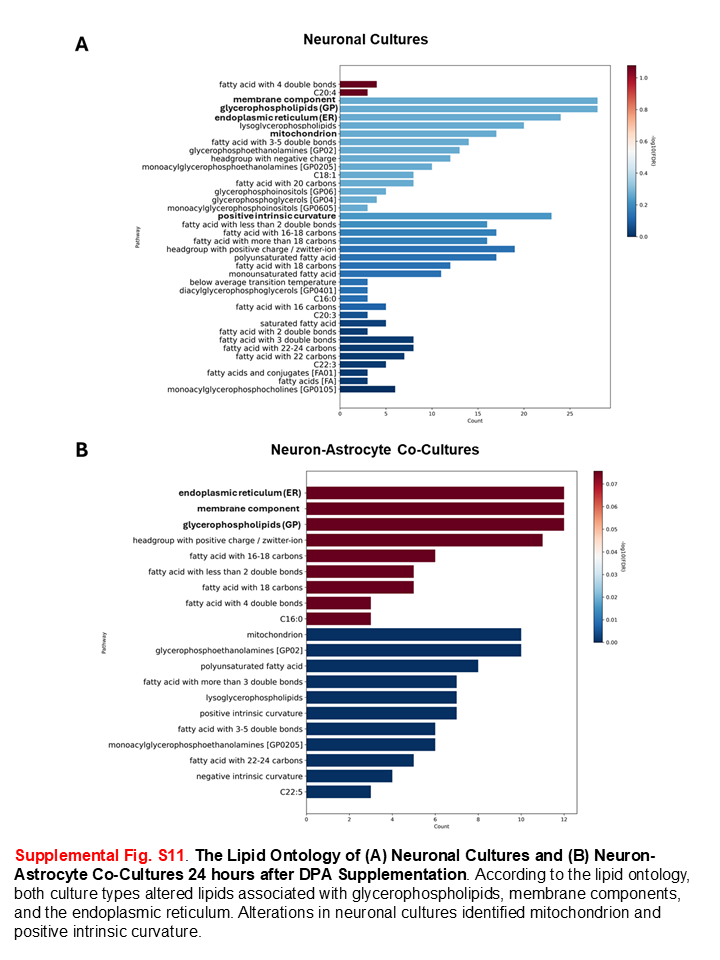

Supplement: Figure S11.TIF [file TASN_A_2644959_SM8041.tif]

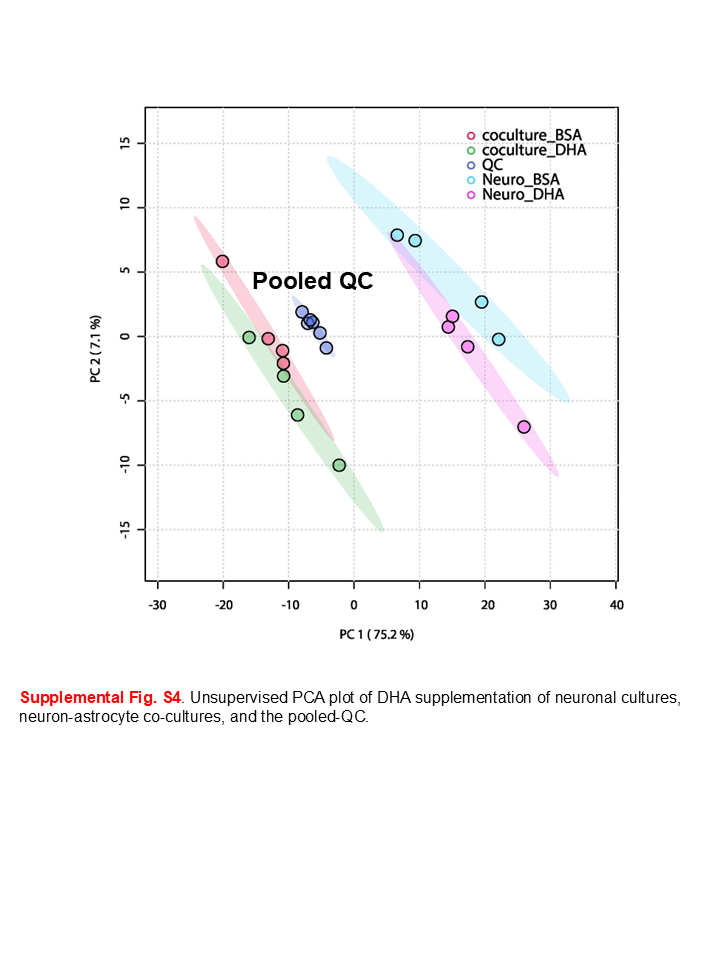

Supplement: Figure S4.TIF [file TASN_A_2644959_SM8040.tif]

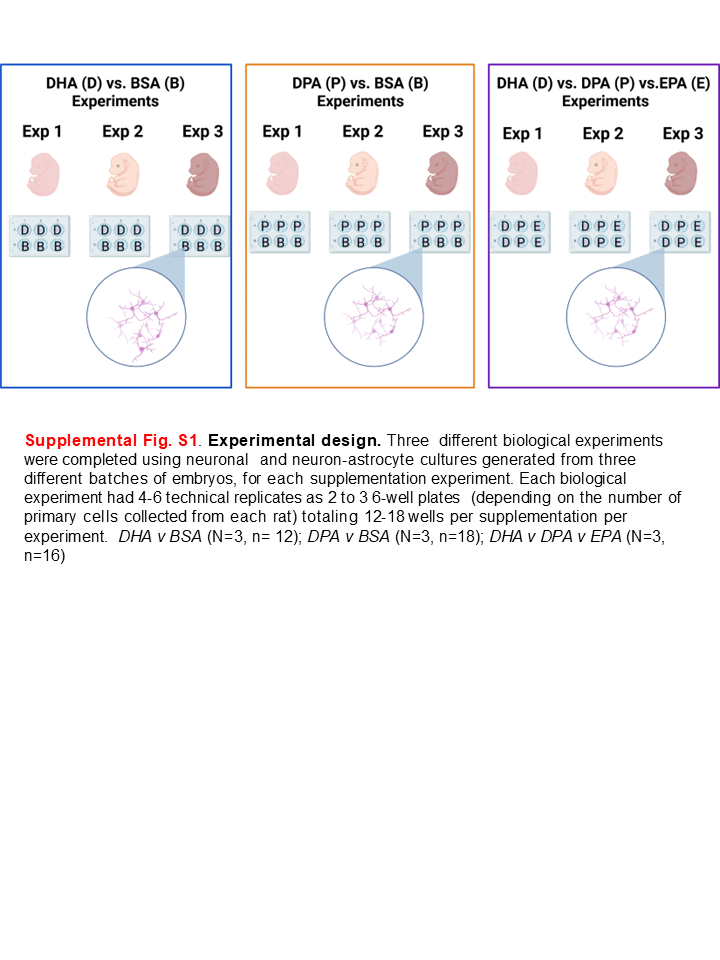

Supplement: Figures S1.TIF [file TASN_A_2644959_SM8039.tif]

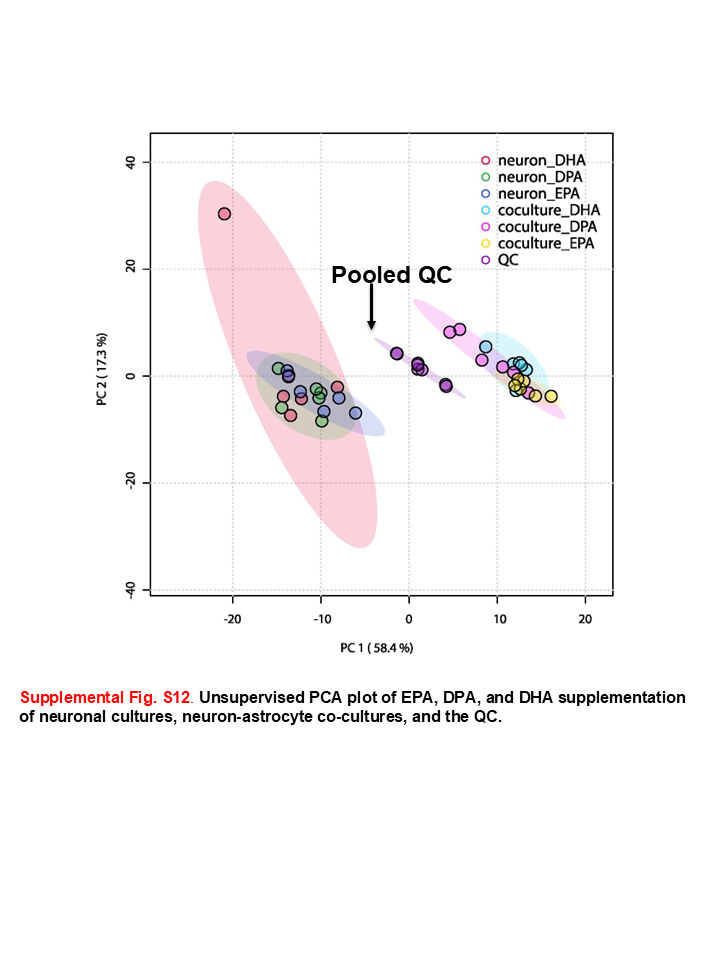

Supplement: Figure S12.TIF [file TASN_A_2644959_SM8037.tif]

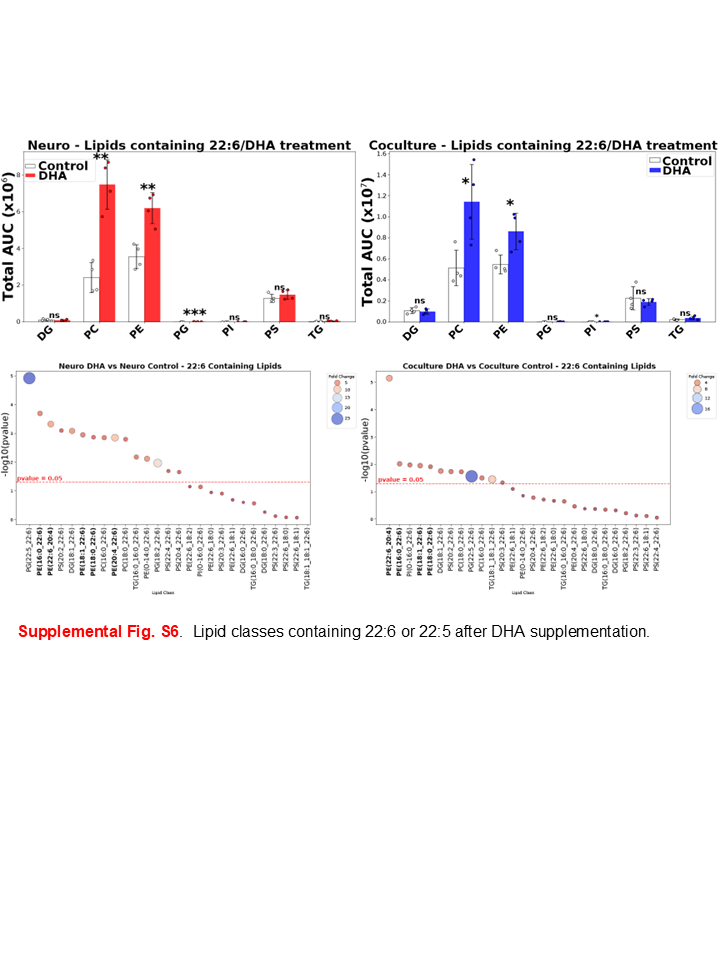

Supplement: Figure S6.TIF [file TASN_A_2644959_SM8036.tif]

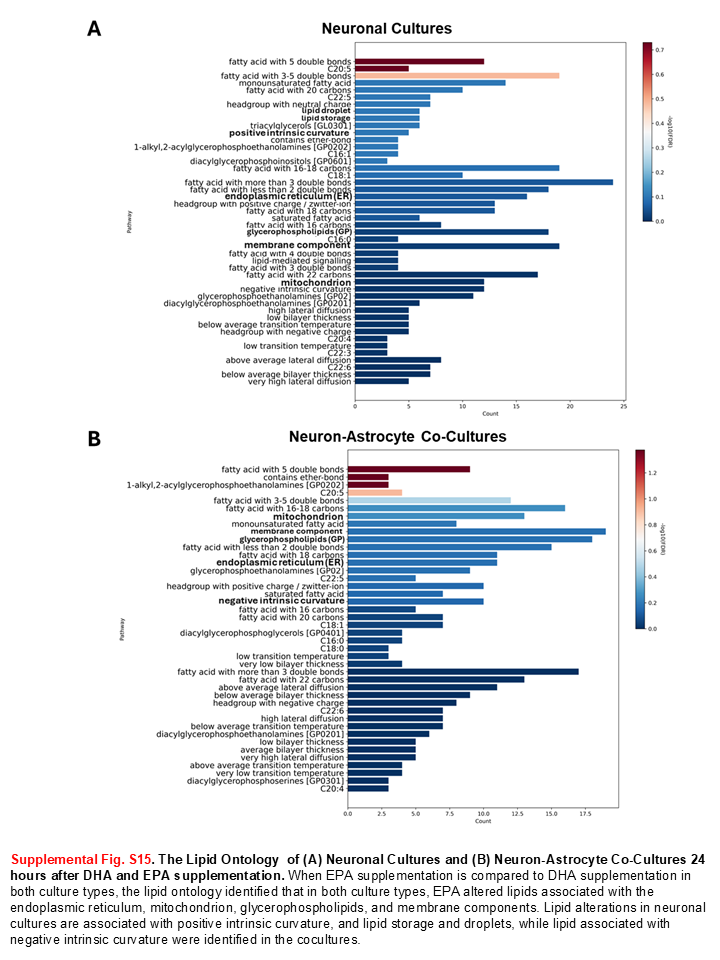

Supplement: Figure S15.TIF [file TASN_A_2644959_SM8035.tif]

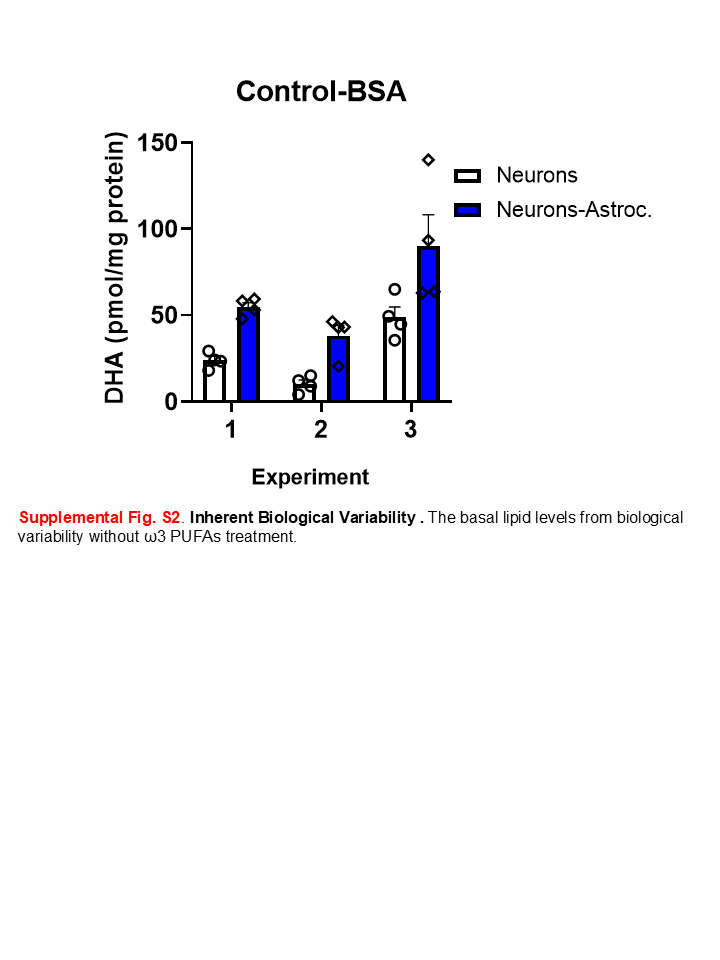

Supplement: Figure S2.TIF [file TASN_A_2644959_SM8033.tif]

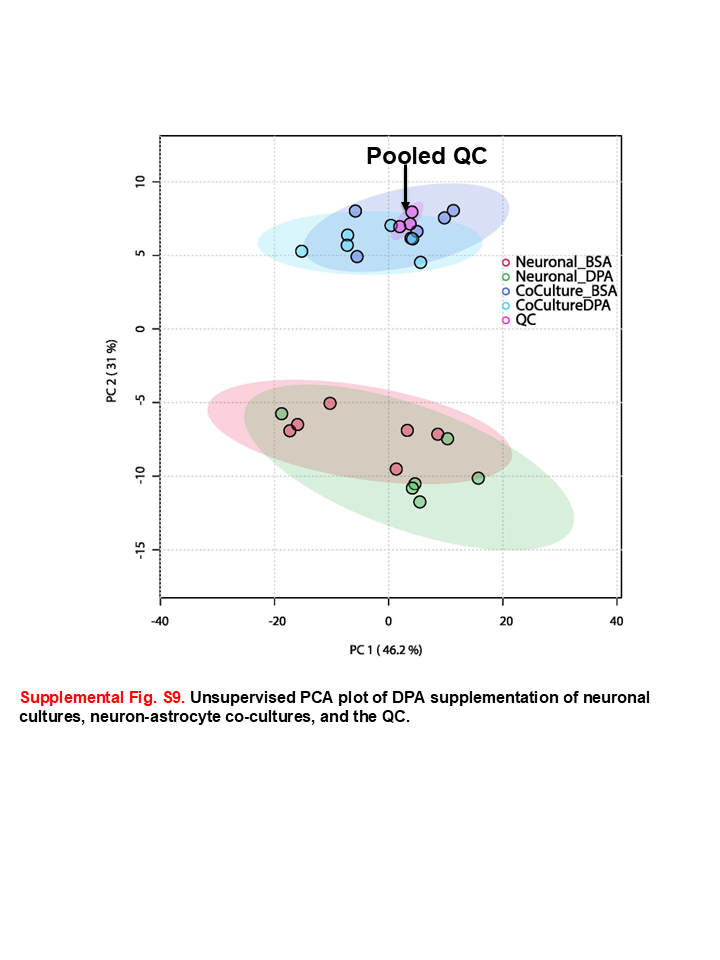

Supplement: Figure S9.TIF [file TASN_A_2644959_SM8032.tif]

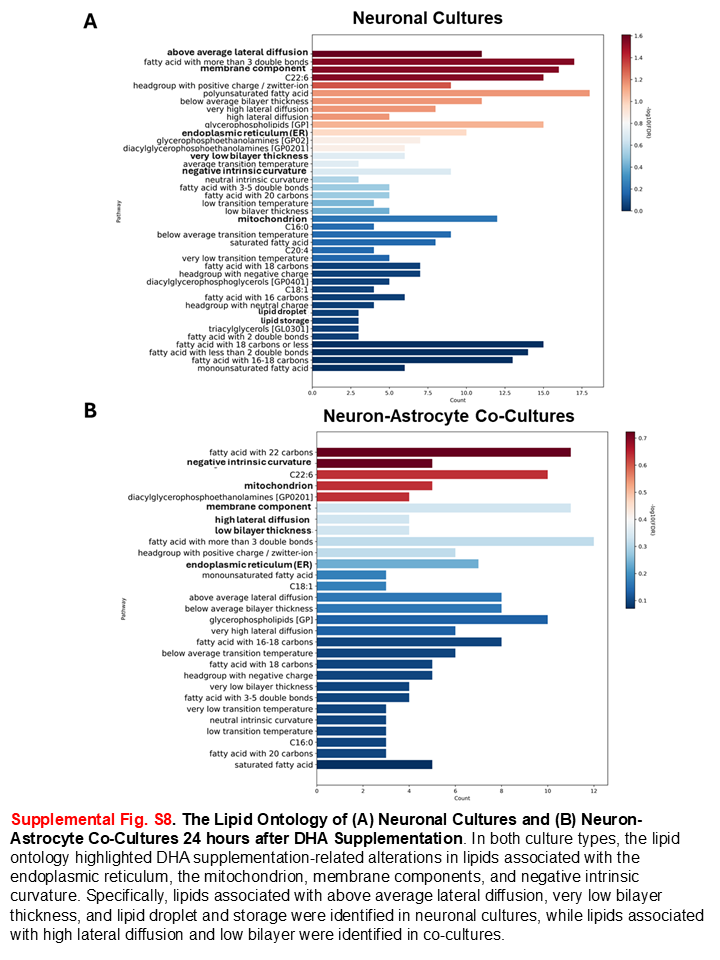

Supplement: Figure S8.TIF [file TASN_A_2644959_SM8031.tif]

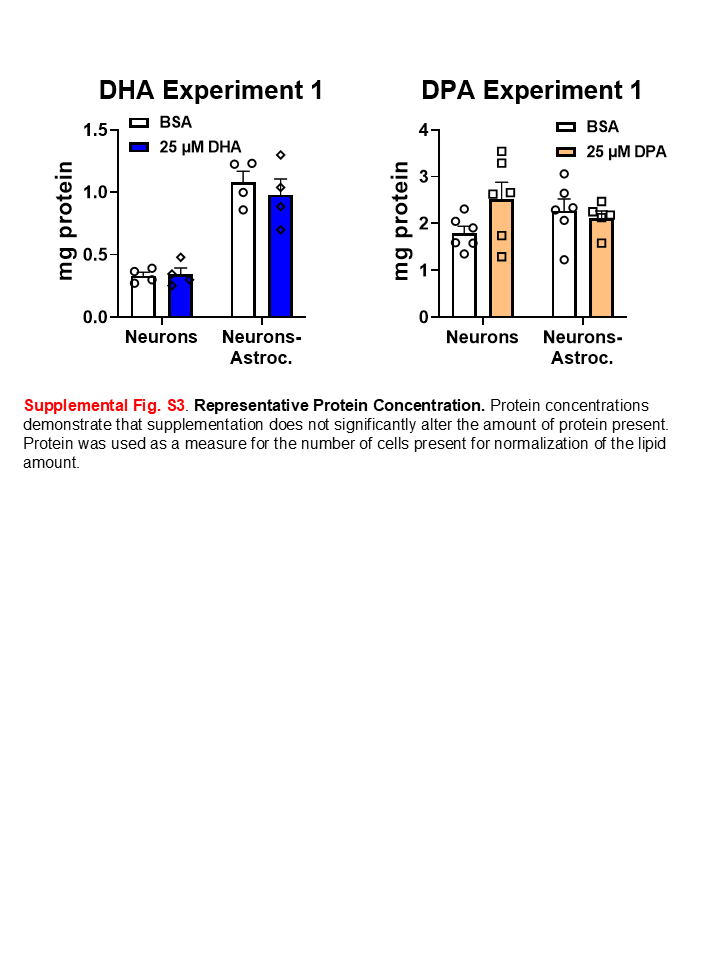

Supplement: Figure S3.TIF [file TASN_A_2644959_SM8030.tif]

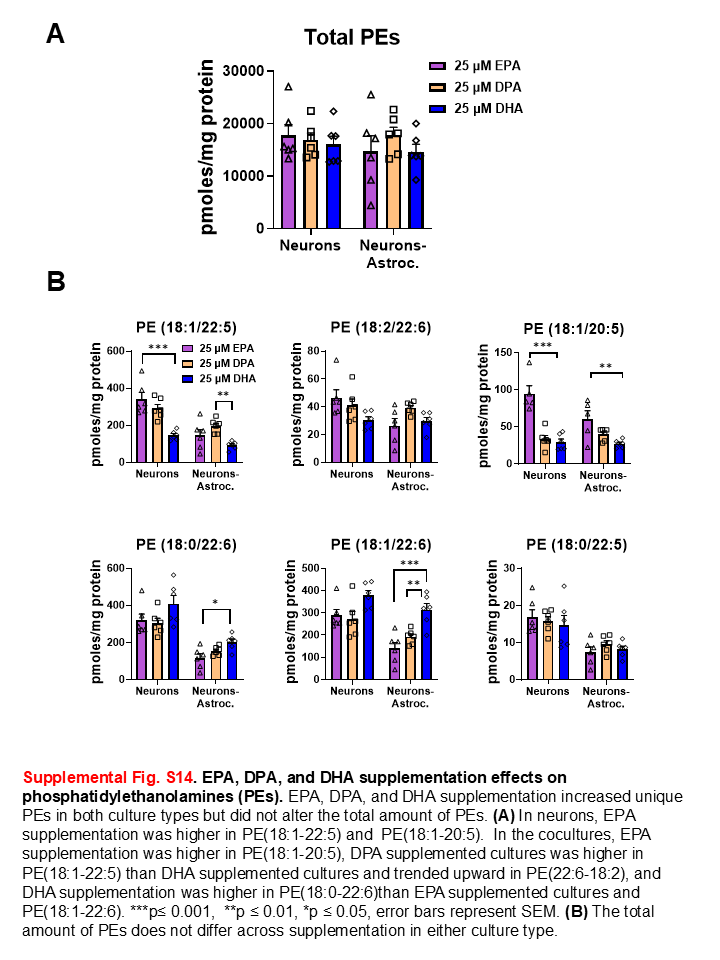

Supplement: Figure S14.TIF [file TASN_A_2644959_SM8029.tif]

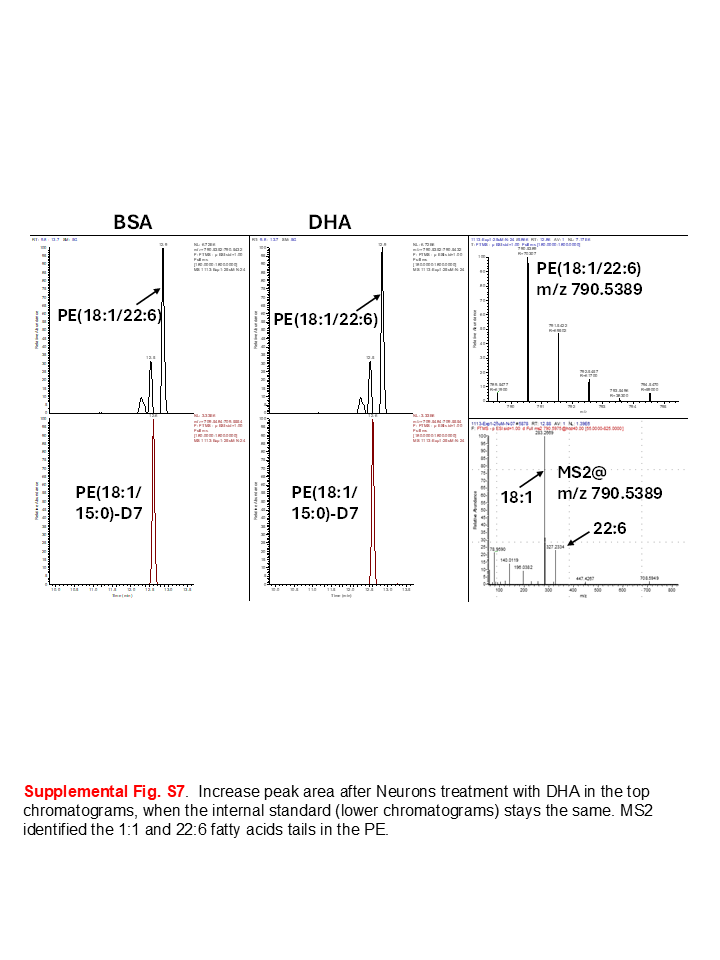

Supplement: Figure S7.TIF [file TASN_A_2644959_SM8027.tif]
